# Spirit of the Foreign Periodicals, &c.

**Published:** 1844-07-01

**Authors:** 


### Spirit of the Foreign Periodicals.

### Miscellaneous Remarks on Hematology.

So much attention is paid in the present day to the study of the Changes of the Blood in different diseases, and so intimately acquainted is this study with some of the most important topics of Therapeutic Medicine, that we offer no apology for again inviting the attention of our readers to a subject that has been pretty amply discussed in the last, and one or two of the preceding numbers of this Journal. There is at present going on, within the economy of the body medical, so curious a process of change, and work of transition from the doctrines of an exclusive Solidism to those of a mixed and modified Humorism, that it becomes most necessary to watch every step in its development and progress, ready to avail ourselves of every well-established discovery, yet guarding our minds against an indiscriminate adoption of every new announcement. In this point of view, the following observations may be read with some profit as well as interest.

The analysis of the Blood of various animals, affected with different forms of inflammatory disease, has invariably exhibited an increase in the normal proportion of its Fibrine. In the *Cachexia aquosa*—known usually by the name of the *rot*—in sheep, the blood is found to be very seriously altered. The proportion of the Fibrine is increased, while that of the Globules, and also of the Albumen in the serum, is very notably diminished below the standard of health. The watery portion or serosity of the latter constituent is much greater than in health; in some cases it amounts to as much as 930 parts in 1000 of the blood. The *Cachexia aquosa* in sheep and the disease of *Albuminuria* in the human subject are the two pathological conditions, in which the proportion of the Albumen in the serum is found to be most remarkably diminished. It is curious however that there is none of this element discoverable in the urine of the animal, when affected with the former malady. But thousands of parasitic Entozoa are usually found dispersed through the substance of the liver and lungs. Moreover, the existence of this malady, even in a high degree, does not exclude the occasional development of well-marked inflammatory action. Under such circumstances, the proportion of the red Globules in the blood continues to be very low; but that of the Fibrine becomes very sensibly augmented, in spite of the general feebleness of the system, and the extreme impoverishment of the blood. It would seem therefore that the increase in the proportion of the Fibrine is always associated with the development of acute diseases in constitutions that are much enfeebled, although the proportion of the red Globules is much lowered beyond the normal standard at the time. It is important to attend to these circumstances, as they serve to point out the general character and tendencies of the Humoral Doctrine of the present day. It is scarcely necessary to say here, that we are indebted for much of our recently-acquired knowledge to the admirable researches of MM. *Andral, Gavarret* and *Delafond.* To M. *Magendie* also we owe not a little. His experiments—which he still pursues with most praiseworthy zeal—have led him to conclusions, that forcibly remind us, in many respects, of the doctrines of the mechanical physicians of the 17th century. Their principles in reference to the mechanism of the Phlegmasiæ are nearly or exactly the same; for, in truth, the theories of the modern pathologist and of his ancient predecessors alike maintain that the process of Inflammation is induced by the red globules becoming unable to traverse the capillary vessels, and by an arrest of the capillary circulation in the parts affected.

The characteristic feature of the old Humoral Doctrine is the unqualified assertion, as the basis of all pathological reasoning, of the vitality not of the blood only, but of all the other fluids of the body. It assumed, as a principle not to be controverted, that they (the fluids) were animated with the same sort of living activity as the solids, and therefore that they, like them, were impressionable from a variety of agencies, both in health and in disease. According to this view of the subject, the Fluids, in virtue of this vital activity, participated in the regular movements of the functions, and might become primarily the subject of their perturbations, as well as the direct agent of the sanative efforts of the system to restore the equilibrium of health. Little engaged with the physical properties of the animal fluids, the physicians of those days paid not much attention to any thing but to their general and external aspect, and almost quite neglected to examine their intimate or elemental composition. Now modern Humorism entirely reverses this mode of procedure. It does not profess to notice or take any account of the inherent activity of the animal fluids; but it confines itself to the task of examining with the most scrupulous minuteness all their physical and chemical properties. We observe the same sort of difference in point of doctrine between the old and modern schools, that has often been remarked in reference to the medical tendencies of the two epochs in question. In the former, the great aim of the physician was to watch the general phenomena of the system, and the more conspicuous manifestations of diffused vitality; while, in the latter, he directs his attention almost exclusively to the profound study of individual facts and special phenomena, and scarcely notices the more general characters of disease. The ancient doctrine was based, as we have already said, exclusively on considerations of the vital activity of the fluids; whereas, the modern one is entirely occupied with studying the material structure of the humours, their forms, and their organic constitution. Without distinctly recognising the independent and proper vitality of the fluids, it is however always to be borne in mind that, as long as they remain within the living body, they possess specific properties or qualities, which cease to exist almost immediately when withdrawn from the agency of the solids, with which they are normally in contact.

If the Fluids of the body possess functions distinct from their physical and chemical properties, we are nevertheless not to deny that there is a bond of union between the functions in question and these properties. Every one knows that the latter are continually undergoing, from a variety of circumstances, numerous modifications which have almost invariably a more or less distinct relation with the conditions of health and disease. For example, the fluids do not exhibit the same characters in respect either of colour, or of density, or of the proportion of their constituent parts, in individuals of different ages, temperaments, sex and so forth; and even the same individual, without becoming positively out of health, is liable to experience vicissitudes of this nature, corresponding with the incessant fluctuations of regimen and the general mode of life. The state of disease very prominently displays the intimate relations that exist between the general characters of the fluids and their peculiar characters as living matter. Who does not well know the density and deep colour of the blood in inflammatory diseases, so very different from what is observed in cachectic and dropsical maladies?—who has not seen its general melting down, so to speak, or sort of aqueous solution, which frequently accompanies the presence of organic lesions, like Cancer, Scrofula, &c.? and surely no one can require to be reminded of the humoral changes that occur in Scurvy, mercurial intoxication, or purulent infection. It cannot therefore be reasonably denied that there is a most close and necessary correspondence between the normal or pathological impressions of the fluids, and the modification of their composition, or their physical changes.

Now it is precisely in this point of view that the Humoral Doctrine of the ancients was most difficult and at fault; it had unravelled with surprising sagacity the physiological and pathological conditions of the fluids, in relation with their impressions and affections; but it failed to detect the circumstances of those impressions or affections which may be reflected in the aspect and structure of the humoral matter. The deficiency in this respect was doubtless owing to the imperfect state of the accessory branches of medical investigation, more especially in the departments of physical and chemical science.—*Gazette Medicale.*

*Remarks.*—We may here very appropriately introduce one or two remarks on the state of the blood in Puerperal Fever, extracted from a recent article on this disease by Dr. *Bouchut.* "One of the most remarkable necroscopic characters," says he, "is the decoloration and liquifaction of the blood, and the small number of the coagula found in the vascular cavities. But, besides these changes, I have observed another alteration, which deserves the especial notice of pathologists, as it affords a strong argument in favour of the *pyogenic* ætiology of the disease. On microscopic examination of the blood of a woman, who died from puerperal fever within 48 hours of seizure, I found, amidst the usual red globules, (which did not seem to be at all altered) a considerable number of larger globules, which appeared to be colourless and to be fringed at their edges: they were either grouped together in clusters of 4 or 10, or they were isolated and detached. The great number of these abnormal globules is opposed to the idea that they were the *white globules,* which always exist in healthy blood. Are we to suppose that pus-globules may become developed within the mass of the blood, without any previous inflammatory action? This opinion was ably supported by *De Haen* and *Home;* and M. *Gendrin* and other pathologists of the present day have endeavoured to prove that the pus-globule is nothing but the ordinary blood-globule somewhat changed."

In his recently published work on Pathological Chemistry (Paris 1843) M. *Lheritier,* the causes of the alterations of the Blood are arranged and described under the following five heads:—

1. Those which vitiate the fluids, that serve for the formation of the blood itself. The chief source of these vitiations is unquestionably the unwholesome nature of the food on which the being lives.

2. Those which in any way disturb the normal operation of the functions of Secretion and Excretion. Of this nature are the changes which are apt to take place in the constitution of the blood, during the prevalence of great heat. It has been shewn by very exact observations that the increase of the atmospheric heat, more especially when the air is at the same time moist and charged with miasmatic effluvia, diminishes very considerably the action of the respiratory process on the blood, and that the Carbonic Acid and other impure elements, imperfectly eliminated in this way, are partly combined to form the Bile—then more abundant than usual, and often vitiated at the same time—and are partly excreted from the system by the surface of the skin and of the intestines. If the functions of these organs—the liver, the skin, and the bowels—which thus compensate for the diminished action of the lungs, are from any cause impeded, the matters, which should otherwise have been eliminated and discharged from the system, must remain behind and accumulate in the blood.

3. Those which contaminate the parts, with which they are brought in contact. To this class belong the cases where putrid matters are introduced into the blood, as well as most probably all contagious diseases.

4. Those which act on the vascular system directly, or indirectly through the medium of the nerves. Such are acids, alkalies and their subcarbonates, metallic salts, various astringents, alcohol, spirit of turpentine, &c.

5. Those which are constituted by the passage of matters formed within the body itself into the circulating fluid. These matters are either the products of secretion, such as Bile, Urine and Milk—although the existence of these substances in the blood has never been perfectly demonstrated—or they are productions which are altogether abnormal and foreign to the organism in a state of health; of this nature are pus, cancerous matter, and various inorganic substances, such as ammonia, &c.

The following explanation of those cases of Jaundice that are apt to be induced by violent mental emotions, and which have usually been supposed to arise from a presumed Spasm of the biliary Ducts, deserves to be noticed.

"This spasm of the hepatic duct," says he, "appears to me to be, if not impossible, at least not at all intelligible; and surely it is much more reasonable to suppose that the cause of the disease under such circumstances, as well as when it has followed injuries of the head, is attributable to the depression of the nervous system—a depression which throws the liver into a state of torpor, so that this organ no longer eliminates and secretes the constituents of the bile from the mass of blood. I therefore conclude that the form of Jaundice in question is owing, not to the resorption of the bile into the system, but rather to a defective or arrested secretion of it. Perhaps indeed there are certain cases of wounds of the head, where the functional condition of the liver has nothing to do with the yellow tint exhibited by the skin: the following case seems to prove this." Here the author relates a case of a man who, after having received a most severe blow on the *left* side of the head from a heavy stone falling upon him, was able to resume his work in three days, although at this time the entire upper extremity of the *right* side, from the shoulder down to the tips of the fingers, exhibited a deep icteric coloration. M. *Lheritier* suggests the following explanation of this phenomenon: "The blood undergoes within the tissues of the body certain transformations, the *ensemble* of which constitutes the process of Nutrition. But what is the agent that ultimately presides over these changes?—the nervous system; well! in the present case the brain had experienced a violent commotion, and, in consequence of this injury, there had ensued a sort of local *anervia,* so that the nutritive transformations were only imperfectly effected; the elements of the blood had not been properly elaborated, dissociated, and combined, and the yellow colouring matter of the bile, contained in this fluid, had therefore become extravasated under the skin."

(This is but a very fanciful conjecture; the yellow discolouration of the upper extremity may have had nothing to do with the formation or excretion of the bile, and was, in all probability, merely of that kind that so generally follows upon bruises and other injuries).

---

### OBSERVATIONS UPON PULMONARY GANGRENE.

The principal results of M. *Boudet's* researches on this subject are these:—

1. Gangrene of the lungs appears to be more frequent in childhood than at any other period of life.

2. It is essentially of the same nature as those other forms of Gangrene that are developed spontaneously, or under the influence of putrescent fever.

3. In the child, Gangrene, when it affects the lung, is rarely limited to this organ; usually several other parts of the body are similarly affected at the same time.

4. The lower and posterior parts of the lungs are most frequently the seat of the disease. We have often occasion to observe that it manifests a remarkable tendency to attack the adjacent organs: thus we not unfrequently find the mediastinum, and the pleura, and sometimes the œsophagus also, involved in the destructive process. It may extend by the mere contact of the sphacelated matter, inducing gangrene in the parts with which it comes in contiguity.

5. Gangrene of the lungs may appear in three different forms: in distinct patches, in nuclei or circumscribed masses, and in a diffused and extensive superficies.

6. It may be circumscribed, and a cure may then be effected in the child as in the adult. In such circumstances, the gangrenous corruption becomes surrounded by an organised pseudo-membrane, so that, in course of time, it is perfectly isolated from the adjacent tissues.

7. Local causes—such as inflammation, the existence of tubercles, &c.—do not seem to have any direct influence on the production of pulmonary Gangrene.

8. Like other spontaneous gangrenes of childhood, this form seems to be always developed under the influence of causes which act on the entire system. Thus, an unsound constitution, shattered health, imperfect or unwholesome nourishment, &c. are observed to predispose to its occurrence. But the proximate cause of the disease is unquestionably a morbid alteration of the mass of blood, consecutive upon Scurvy, Measles, Scarlet-fever, and such like maladies, and which is characterised during life by the appearance of purple spots on the surface, softening of the gums, hæmorrhage from various parts, &c. and after death, by patches of ecchymosis, and sanguineous suffusion, and by the extreme fluidity or dissolved state of the blood. In a chemical point of view, this state of things is most probably the result of a diminution in the proportion of the fibrine, and an excess in that of the alkaline ingredients of the blood.

9. The usual symptoms of Gangrene of the lungs are dulness on percussion over the affected part, resonance of the voice, mucous or gurgling râle, fætor of the breath, greenish colour of the sputa, and a peculiar expression of the features, that cannot be well described by words.

In reference to the treatment of this disease, M. *Boudet* makes the following remarks:—"It is almost always after Measles or Scarlet-fever that gangrene of the lungs has been observed to occur. Therefore, as a means of prevention, the first step to be taken should be to keep, as far as in our power, children from being exposed to the contagion of the disease. The children who may be affected with it, should be carefully separated and kept apart from the other inmates of an establishment, like that of the Hôpital des Enfans. Moreover, as it has been shewn by the most satisfactory researches, that the Exanthemata in question are generally more severe and complicated in children that have not been vaccinated, I should most strongly urge upon all parents, as well as medical men, a more punctual attention to this most necessary precaution."

Whenever, during the progress of Measles or Scarlet-fever, there is observed any tendency to the occurrence of hæmorrhage, or sponginess of the gums, or purpural eruption on the skin, the physician should diligently examine the state of the lungs; for it is in such a state of the system that pulmonary Gangrene is generally developed. The patient should immediately be ordered the free use of a beverage made with lemon-juice or with sulphuric acid: the affected parts of the mouth should be touched with nitric or muriatic acid; acid and antiseptic gargles should be used frequently, and the body and limbs should be sponged with an aromatic acid lotion. Dr. *Boudet* assures us that he has seen, in the Salpetrière, many cases in the most advanced stage, and seemingly in a desperate condition, recover under the diligent employment of acid medicines, internally and externally administered. The acids serve to counteract the excessive Alkalinity of the Blood, while the cordials give tone to the system, and enable it to resist the tendency that exists to the loss of the Fibrine. The application of a blister or a sinapism to the legs is often a useful adjuvant; and in many cases decoction of bark, with port wine and other tonics, may be advantageously exhibited. Some physicians have spoken well of the use of the Chlorurets, given internally as well as freely applied to the surface of the body and to the gums. As it is the lower and posterior part of the lungs that is generally affected, the position of the patient should be frequently altered, so that the seat of the disease may not always be in a depending position.—*L'Experience.*

---

### MICROSCOPIC ANATOMY OF TUBERCLES.

The following are some of the most important conclusions of an elaborate Memoir, that was recently communicated to the Academy of Sciences.

1. The constant Microscopic Elements of tubercles are these: *a*, molecular granules; *b*, a hyaline interglobular substance; and *c*, the proper corpuscles, or globules of tuberculous matter. These globules contain a number of molecular granules, but no distinct nuclei. They are not affected by water, ether, and the feeble acids; but they are dissolved by the strong acids, as well as by ammonia and caustic potash.

2. The opinion of certain pathologists, that tuberculous deposit and its globules are only modifications of purulent matter, is contradicted by the result of microscopic inspection; the differences between them are strong and decided. The corpuscles of the latter are considerably larger, of a regularly spherical shape, and contain from one to three nuclei: they are, moreover, usually free and isolated; whereas those of tuberculous matter are, especially in the crude state of tubercles, closely joined together. The globules of Cancerous matter are twice or even four times as large, and they contain a nuclei, in which again from one to three nucleoli are often observable.

3. In Sarcocele, and also in Scirrhous and Encephaloid tumors of the mammæ, we not unfrequently find a yellowish cheesy-looking substance, which much resembles genuine tuberculous matter; but a careful examination with the microscope clearly shews that it consists entirely of globules of Cancer infiltrated with fat.

4. When tubercles soften, their interglobular substance liquifies, the globules separate from each other, and may then, by absorbing a certain portion of the fluid, become larger: this change does not constitute an increased growth, but, on the contrary, the commencement of the process of decomposition.

5. The pus, which is found blended with the softened tuberculous matter, is supplied by the surrounding tissues, and is by no means the result of any transformation of the matter itself; but the pus, it must be confessed, quickly alters it, and renders its elements much less easily recognizable.

6. The globules of softened tubercles become ultimately dissolved in a granular fluid, and thus the *ramollissement* of their substance passes fairly to the state of diffluence.

7. The cretaceous condition of tuberculous matter presents under the microscope the appearance of amorphous mineral granules, blended often with crystals of cholesterine and colouring matter. A part of the tuberculous globules is then removed by absorption, while the other portion remains for a long time in an unchanged condition.

8. Occasionally we find in tuberculous deposit corpuscles of fat, melanosis, greenish-coloured globules, and crystals which have the form of those of the ammoniaco-magnesian phosphate. Besides these admixtures, we may find, blended along with them, the elements of inflammatory and suppurative action, and various sorts of epithelial exsudation; all of which tend to modify the essential microscopic features of the tubercles.

9. The seat of tubercles in the lungs is usually the inter-vesicular elastic cellular (or areolar) tissue: sometimes, however, they are secreted into the air-vesicles themselves, and into the capillary bronchial tubes.

10. The semi-transparent grey granulations of the lungs are composed of tuberculous granules, interglobular substance which is more abundant and more transparent than in the yellow tubercles, and of pulmonary fibres more or less altered in their appearance. They are not invariably the "point de depart" or primary condition of the miliary yellow tubercles; as these latter are sometimes developed as *such*, from the very commencement of their deposition.

11. Microscopic examination most decidedly exposes the fallacy of the opinion, that the grey granulation is the product or effect of inflammatory action.

12. A vomica or tuberculous excavation is in every respect analogous to a tuberculous ulcer of the skin or intestinal canal; it is not necessarily preceded by, or the result of, the suppurative process. As a general remark, it may be asserted that Phthisis is accompanied with an ulcerative diathesis.

13. *The* fluid of pulmonary excavations contains the following elements: *a*, tuberculous matter, with globules which are either much more turgid than usual, or are altogether diffluent; *b*, globules of pus sometimes in small quantity; *c*, puoïd globules; *d*, granular globules; *e*, globules of mucus or of muco-pus; *f*, blood globules; *g*, pulmonary fibres; *h*, black pigment; *i*, epithelium shreds; *j*, crystals; and *k*, globules of fat. (Surely there is a good deal of hair-splitting nicety in such an enumeration as this.)

14. The cavity of a Vomica is lined with a pyogenic membrane, the formation of which may be regarded as a Curative effort of Nature to isolate the cavity from the surrounding tissue, and thus to favour its cicatrisation. The cicatrisation is in many cases promoted by a new secretion of fibrous matter, and occasionally also of a chalky deposit at the same time.

15. The thickening of the pleura over the seat of tuberculous deposit is the result not of inflammation alone, but also of an augmented nutrition or hypertrophe, in consequence of the increased flow to it of the blood, which formerly permeated the (now obliterated) capillary vessels of the surface of the lungs. It thus becomes a supplementary organ of circulation in Phthisis, and serves to increase the Anastomoses with the Aortic Circulation by its intimate adhesions with the parietes of the chest.

15. The Liver is often the seat of extensive tuberculous deposit; and this lesion may readily be mistaken for Cancerous transformation. The distinctive microscopic characters are most to be trusted to in the pathological diagnosis of such cases.

16. The fatty degeneration of the Liver and Heart—so accurately described by M. *Bigot*—exhibits a tendency to the internal deposit of fatty matter in Phthisis, while all the fat of the external parts of the body is at the same time entirely absorbed.

17. We occasionally find a quantity of tuberculous deposit in old membranous deposits on the Pericardium. In a case, where the Pericardium adhered firmly to the surface of the heart and all the surrounding parts, numerous Anastomoses between the ramifications of the Coronary vessels and those of the surface of the lungs were found to have been established.—*Gazette Medicale*

---

### Professor Forget on Adhesions of the Pericardium.

The substance of this active Professor's remarks on this subject is contained in the following observations, with which he closes a lengthened narrative.

1. Pericarditis often terminates in general or partial adhesions of the heart with its enveloping Capsule.

2. These adhesions seem to succeed more especially to the *dry* (without effusion) form of the disease in question.

3. They are a potent and very frequent cause of disturbance of the heart's actions.

4. The disturbances thus induced are usually the more serious and dangerous, in proportion as the adhesions are the more recent and the more extensive.

5. The characteristic signs of recent general adhesion are the force, the tumult and confusion of the beats of the heart; the frequency, smallness, inequality and irregularity of the pulse; the dyspnœa, precordial anxiety, and tendency to fainting; the serous infiltration of different parts; the pulmonary congestion, visceral engorgements, cyanosis, &c.

6. The sign, so much insisted upon by some medical men—viz: depression of the epigastric region during the contraction of the heart—has not been observed in a single case of pericardial adhesion in our practice; and yet we have occasionally recognised its existence in some other diseases of the heart.

7. Not any one of the symptoms enumerated above, however valuable in a diagnostic point of view, is pathognomonic of, or exclusively peculiar to, adhesions of the pericardium. It is moreover rare to find them all combined in the same case.

8. Adhesion of the pericardium may prove fatal in the acute state; or in the chronic state by inducing some other morbid lesion, such as Hydrops pericardii, &c.

9. The more ancient that the adhesions are, the less is life menaced by their existence: it may even so happen that scarcely any appreciable embarrassment is caused thereby to the movements of the heart.

10. The only advantage, so to speak, of general adhesions is that they serve to diminish, or even altogether to abolish, the tendency to future pericarditis, just in the same manner as the provoked adhesion of the tunica vaginalis prevents the recurrence of common Hydrocele.

11. The presumed relations of adhesion of the pericardium with Hypertrophe of the heart are certainly very reasonable; but the presumption awaits the sanction of demonstration.

12. The circumstance of adhesion being a frequent termination of pericarditis is an additional motive for prompt and vigorous treatment to subdue the inflammation, when discovered.

13. The diagnosis of the formation and existence of pericardial adhesions may be useful in many respects; it may enable the physician to arrest in time the progress of the adhesive inflammation, or obviate the aggravation of those accidents which adhesions are apt to induce in various maladies, as well as in a state of health.

---

### ON THE ANIMALCULAR CONDITION OF THE BLOOD IN DOGS.

M.M. *Gruby* and *Delafond* have recently addressed a second communication to the Royal Acadamy on the verminous alteration of the blood in dogs, by the presence of hematozoary animalcules of the genus *Filaria*. The principal results to which they have arrived are,

1. The proportion between the number of dogs whose blood is verminous, and of those whose blood is not so, seems to be nearly as 1 to 50.

2. Dogs with verminous blood enjoy very good health, and seem to retain all their instinctive faculties.

3. The blood in such animals is usually redder and more serous than in the ordinary condition.

4. Regimen, the sort of food, exercise, losses of blood, &c. appear to have no influence whatsoever on the number, form, and movements of the *filariæ*.

5. Two *decilitres* of verminous blood, which had been defibrinated, were transfused into the veins of an animal whose blood was not verminous, without giving rise to the production of these animalcules in the case of the latter, during eight days after the operation. But when a *litre* of the verminous blood had been injected, *filariæ* were discoverable in the blood of the animal operated on; yet without any injury to its health being induced.

6. The *filariæ* of the blood are not found in the excrementitious matters of the animal or in any of the other humours, such as the saliva, bile, urine, &c.; nor in any purulent discharge; nor yet in the chyle or lymph. Neither do they exist in any of the simple or compound tissues of the organism.

7. The verminous condition of the blood seems to be constant and durable in certain animals, and to have no injurious influence on the state of their health and faculties.—*Comptes Rendus.*

---

### M. Orfila's Reply to certain Misrepresentations.

1. "I had established," says this distinguished Toxicologist, "in my first Memoir on Arsenic published in January 1839, that a notable quantity of the poison was discoverable in the blood of a dog, within one hour and twenty-five minutes after it had been received into the stomach.

"In the course of last February, M. *Chatin* announced to the Academy of Sciences that, not only in eight cases of dogs poisoned with arsenic and antimony he had been able to obtain distinct traces of both metals from the blood, but also that he had found traces of the latter metal in the blood of human patients, who had been taking large doses of emetic tartar.

"What then was my astonishment on reading, in an account of a recent meeting of the Academy, the following passage from a note of MM. *Flandin* and *Danger:* 'Whatever be the moment when the blood, destined for examination, be drawn from an animal that has been poisoned by any metallic substance, we never find the poisonous element in the blood.' To this assertion I shall only reply that I am ready at all times to prove to the Commission now sitting, that it is utterly inconsistent with truth.

2. "In the same note of these gentlemen we also meet with the following very erroneous statement: 'Hitherto, in judicial examinations, it has been more especially in the blood and in the heart that the experimenter has sought for the existence of poisonous substances.' Now this assertion is quite as untrue as the preceding one; for I will venture to assert that they cannot adduce a single experiment in which the examination was limited to the blood and to the heart, when the liver and some other organs could be obtained.

3. "In another letter which he has recently published, M. *Flandin,* in order the better to establish the claims which, he thinks, he has to the discovery of the important fact, that poisonous substances are found on post-mortem examinations much more abundantly in the parenchymatous substance of the Liver than in other organs of the body, pretends that in my laboratory experiments I have usually analysed, at one and the same operation, the liver, spleen, kidneys, heart, and lungs. If such were indeed the case, I should be ill entitled to claim to myself the *idée-mere,* to which I at present allude. But this assertion of M. *Flandin* is as utterly destitute of foundation as the others, and its inaccuracy may be readily disproved by any one who will take the trouble of reading my writings with ordinary attention.

"The experiments 6, 10, 16 and 17 of my first Memoir on Arsenic abundantly shew that I acted on each of the principal organs *separately,* and not conjointly. For example, in the report of the 16th, it is stated that 'the brain contained scarcely any traces of arsenic; they were rather more distinct in the lungs; and still more so in the heart and kidneys. The liver and spleen contained more than any of the other viscera.' In examining the body of *Soufflard,* I acted separately on the liver, the kidneys, and the lungs; and I can shew to the Commission, if they should desire it, the specimens of arsenic which I extracted from each of these viscera.

" But it is especially in my Memoir on Antimony, read before the Academy in March 1840, that the fact now under consideration is dwelt upon in such a manner as not to leave the slightest doubt. For, of *six* experiments described in that Memoir, *five* were performed by treating the organs separately; and I then distinctly stated, as the result of my observations, that the liver and kidney contained much more of the metal than any of the other viscera.

" In reply to the reproach that M. *Flandin* directs against me, of considering the animal body as a mere sponge that is apt to become passively charged with certain substances *floating* through its channels, I have only to say that I entirely adopt the theory of absorption that is advocated by such physiologists as MM. *Fodera* and *Magendie.*"—*Comptes Rendus.*

---

### AWARD OF PRIZES AT THE ACADEMY OF SCIENCE.

The Commissioners, appointed to examine the works on Medicine and Surgery that had been sent in for competition, and to decide upon their claims to Academical distinction, were MM. *Breschet, Double, Serres, Dumeril, Magendie, Roux, De Blainville, Pariset, Velpeau* and *Andral* (the reporter)—men certainly that the French metropolis may justly be proud of in the present day, and whose high character is an abundant guarantee alike for impartiality and for professional judgment.

Twenty-six authors appeared on the field as candidates; and out of these the Commissioners had to select the names of those who, in their opinion, had contributed most prominently to the advancement of medical or surgical science.

The following summary of their report will be read with considerable interest.

1. The surgical operation for the Cure of Squinting was introduced into surgery some years age. Devised and originally proposed by M. *Stromeyer*, it was performed for the first time—on the living subject at least—by M. *Dieffenbach*, at Berlin, in 1839. Within a short period, he repeated the operation a great number of times; and, ere long, it was pretty generally adopted in every country of Europe. Various changes and modifications were, as a matter of course, suggested and adopted by different operators. The results obtained have been found to vary not a little. In consequence of several surgeons submitting the results of their experience to the Academy of Sciences, as claims for the *Monthyon* prize, it necessarily became an early subject of enquiry for the Commissioners to examine and determine upon the merits of the operation in question. At that time (1841), they felt themselves unable to come to any very decided opinion, and they therefore postponed the expression of their judgment to a future day. Since that period, the operation has been much more attentively studied, and its true value has been better ascertained; and the Commissioners therefore feel themselves warranted in now declaring their deliberate opinion, that it is justly admitted into the domain of surgery, as one of the recognised achievements of the art. For this reason they have proposed that the sum of 6000 francs be awarded, in equal proportions, to MM. *Stromeyer* and *Dieffenbach*—the one for having originated, and the other for having first performed, the operation of Myotomy for the cure of Squinting.

Respecting the modifications and alleged improvements, that have been suggested by various communicants, the Commission feels itself quite unable to determine their respective merits.

2. Under the title of " Iconographie d'Anatomie Chirurgicale et de Medecine

Operatoire," MM. *Bourgery* and *Jacob* have published a work, that cannot fail, in the judgment of the Commission, to render essential services to Surgery. The plates, which are admirably executed, represent every surgical operation that is ever performed with surprising truthfulness and exactitude. A prize of 5000 francs is bestowed upon the authors.

3. If it is incontestable that the advancement of medicine is, in a great measure, due to the care with which the various changes, produced in different organs by disease, have been studied, we cannot but receive with favour every rational attempt to represent these changes in a palpable form; for it will be admitted by all that mere description, however accurate, will seldom suffice to give a just idea of them. In this respect, the artificial specimens of pathological Anatomy prepared by Dr. *Thibert* are entitled to most honorable mention. He has succeeded in reproducing, with remarkable fidelity, a variety of morbid alterations and changes of structure. Having first represented them by coloured designs, he moulds them, and then obtains casts with a material analogous to 'carton-pierre,' which is afterwards painted as near to nature as possible. The pathological characters as to form, relief and colour, are thus given with wonderful accuracy. The Commissioners have examined many specimens representing various affections of the skin and mucous membranes, especially the alteration of the intestinal follicles in Typhoid fever; also numerous diseases of the brain, liver, lungs, and osseous system; and they have been invariably struck with their remarkable fidelity and truth. They have moreover satisfied themselves that these specimens of art remain uninjured by exposure to air. As many copies may be taken from the mould as are required, and their price is very much less than similar representations in wax. It must therefore be obvious that they will be exceedingly well suited for the purpose of lecturing, and so forth. A reward of 4000 francs has been granted to Dr. *Thibert.*

4. The sum of 3000 francs is awarded to M. *Longet,* to mark the Commissioners approval of his recent work, entitled, "Anatomie et Physiologie du Systeme Nerveux de l'Homme et des Animaux Vertebrés, avec les Observations Pathologiques relative au Systeme Nerveux de l'Homme." The author first traces with great accuracy the structure and functions of the nervous system in all the classes of Vertebrated animals, and he then applies the knowledge so acquired to the elucidation of various pathological conditions in the human body. He has collected a vast number of facts, bearing on the history of Diseases of the Nervous System, and which hitherto have been scattered over a multitude of different works. In this manner he has been enabled, at the expense of great labour, to test their comparative value, and to correct the conclusions of a more partial and imperfect observation. He has thus succeeded in advancing our knowledge of the semeiology of Nervous Diseases—a nosological groupe, however, which still exhibits many difficult and perplexing subjects of investigation to the pathological enquirer. In acknowledgement of M. *Longet's* meritorious researches, a sum of 3000 francs is bestowed upon him.

5. Dr. *Valleix* has published a work on Neuralgic disorders, which is well calculated to throw light on the history, and to improve and methodise the treatment of these troublesome affections. He has analysed, with no common sagacity, the results of his predecessors' observations in this department of pathological enquiry, separating the wheat from the chaff, the practical and really instructive matter from what is merely speculative and fanciful. Hitherto, authors have attended almost exclusively to certain forms of Neuralgia, more especially when it affects the facial and the femoro-popliteal nerves; but M. *Valleix* has embraced a much wider field of enquiry, and examined with great accuracy the painful affections of the cervico-occipital, the dorso-intercostal, and the lumboabdominal nerves also. The isolated production of pain in certain circumscribed points of nervous cords is a circumstance in the history of Neuralgia, which—in the double point of view of diagnosis and treatment—had not attracted the attention which it deserves, before the publication of M. *Valleix's* work. A sum of 2000 francs is awarded to him.

6. Among the works which deserve honorable mention, the Commission have much pleasure in signalising the Memoir of M. *Amussat,* which he presented to the Concours for the Monthyon prize of 1842. It contains a summary of a great number of experiments,—accompanied with numerous illustrative drawings and pathological specimens—undertaken with the view of shewing,

*a.* That a wound made in an artery is capable of becoming so firmly and solidly cicatrised that it is not necessary, in a great number of cases, to tie the vessel with a ligature, but only to compress it with a moderate degree of force.

*b.* That the coagulation of the blood—which, on wounded surfaces, is effected in the ends of the divided capillaries, in consequence of the retraction of their middle or elastic coat and also of the infiltration of blood into their cellular tunic—gives rise to the formation of a sort of solid 'mamelons,' which may materially aid the surgeon in discovering the arteries, which it is his object to tie.

*c.* That when a large artery is wounded, the surgeon may almost always distinguish, in the middle of the coagulum that is formed by the effused blood, a sort of crater or well which has a somewhat different colour and consistence from those of the surrounding parts, and which may therefore serve as a guide to direct the tenaculum straight to the wounded point of the vessel.

*d.* That various sorts of aneurismatic swellings may be produced in animals, by inflicting wounds in different manners on their bloodvessels.

While the Commissioners are willing to render every justice to the zeal and ability with which M. *Amussat* has carried out his researches, they feel bound to say that, in their opinion, some of the practical conclusions, which he has drawn, are by no means satisfactorily established. For this reason, they are induced to make honorable mention of his labours, and to solicit him to persevere in their completion.

7. The work of MM. *Serrurier* and *Rousseau,* on the diseases of the air-passages in man and the lower animals, merits an equally honorable notice. The researches of these gentlemen in the Comparative Pathology of the diseases in question deserve especial commendation, for they tend to throw considerable light on their history and treatment. MM. *Serrurier* and *Rousseau,* it is hoped, will find, in this public mention which is accorded to their labours for the second time, in consequence of the additions which they have recently made to their work, a fresh encouragement to the prosecution of their interesting researches.

8. Lastly, Dr. *Philippe Boyer* is entitled to honorable mention for his work on the treatment of Ulcers by compression with strips of gummed diachylon plaster, and for his truly praiseworthy exertions in extending the benefits of his method of treatment to a vast number of poor patients.

9. The physiological prize of 2000 francs is awarded to M. *Laurent* for his admirable and most instructive Researches on the common green Hydra.—*L'Experience.*

---

### ON THE TENDENCIES OF COTEMPORANEOUS SURGERY.

The following very judicious remarks are from the pen of M. *Janson,* ex-Surgeon in Chief of the Hôtel Dieu at Lyons.

" For a long time, the opinions of *Dessault* exercised great influence and a most imposing authority in all the schools of surgery, which did not escape the impress of the seal of that age, when physical and mathematical sciences everywhere so much predominated. Without alluding to the art of war, which then occupied the attention of the whole world, it is worthy of notice that the study of Chemistry and Natural History quite absorbed the minds of all educated persons: these branches aspired to the supreme rank in general science; and Operative Medicine sought to assume a like pre-eminence in the medical hierarchy. Surgery, thus separating itself completely from the other branches of medical science, was then unwilling to recognise anything but material facts, geometric demonstrations, and mechanical processes and contrivances; the methods which it employed, so far from being more simplified, became every day more and more complex by a 'luxe' of instruments and apparatuses, which were in reality good for nothing else, save only to encumber our professional *armamentaria.* Surgeons knew, or seemed to wish to know, nothing but Anatomy and Surgery; every one sought to distinguish himself in these two branches—the most important, it is true, but not the only positive or essential, departments of the Healing Art. Then was truly the epoch of Anatomical Surgery. If we open the works of *Sabatier, Lassus, Percy, Pelletan, Boyer,* and *Petit,* we find in every page of their writings that the classical surgery of this period was founded almost exclusively on exact, and even minute, Anatomical data; and we seldom or never meet with any allusion to medicine, any happy application of physiological truth, or any comprehensive therapeutic views apart from mere mechanical instrumentation.

" The fine genius, however, of *Bichat,* prepared the way for an epoch of reform. Without abandoning the path trodden by his Cotemporaries, and without renouncing the study of anatomy and its immediate application to operative surgery, this celebrated man clearly perceived that it was high time to bring medicine and surgery more closely together, and no longer to isolate the material study of our organs from the appreciation of their physiological phenomena.

" What *Bichat* had partially foreseen and pointed out, some of his cotemporaries were not long of carrying into effect, to the great advantage of the art, and in the genuine spirit of the unity and indivisibility of medical science. The young surgeons of this period were, every one of them, either public teachers, or authors of treatises on Physiology; and it was not until they had acquired a reputation as anatomists and physiologists, that they devoted themselves, with an indefatigable ardour, to the pursuit of Operative Medicine.

" Thus *Dupuytren* published at the same time his 'Theory of the Fractures of the Fibula,' and his beautiful 'Report on the Yellow Fever and on Contagion.' If, on the one hand, he invented his Enterotome, on the other hand, he made us acquainted with his researches on Respiration, on Absorption, and on the Movements of the Brain; and thus it was that, if he was the first Operator of his age, he was not less distinguished as the founder of pathological anatomy.

" *Richerand,* too, discussed at one and the same time the subject of fractures of the thigh-bone, and the medical treatment of Ulcers; and, if he examined with great zeal the best plan of performing partial amputation of the foot, and was the first to excise diseased ribs, it is equally worthy of notice that he has left us excellent Monographs on Scurvy and on Scrofulous Affections. He is the author, every one knows, of a most interesting work on Physiology, on the one hand; and of an elaborate Treatise on Surgery, entitled 'Nosographie Chirurgicale,' on the other.

" *Delpech* and *Leveillé,* in their Medico-Chirurgical Researches on Necrosis, Aneurisms, Hospital Gangrene, Pyogeny, and Gymnastics applied to the general treatment of diseases, have trodden the same path, and contributed to the production of like results.

"Our illustrious preceptor, Professor *Roux*, in his Miscellanies of Surgery and Physiology, has discussed the various topics of fibrous tumors of the Uterus, and the phenomena of Continuous Inflammation, of Wounds and Sympathetic Affections, of Hernia and the process of Secretion. It was he who devised and first performed the operation of Staphyloraphy; and to him we owe an excellent memoir on the diagnosis of chest complaints by abdominal pressure.

"These distinguished men may be considered as the leaders or chiefs of the Epoch of what may be called Physiological Surgery—which was dominant in the schools, when the publication of *Broussais*' celebrated work, 'Doctrines Medicales,' came to give another impulsion to the system of reform, which was at this period rapidly advancing to its fulfilment.

"The start being once given, every one seemed ready to reply to the appeal that was now made to him. Medical education became much more general and complete, and all surgeons manifested an ardent wish to escape from the narrow circle in which they had been so long confined themselves. They now better understood and appreciated the many resources of nature; and, in cases apparently the most serious and desperate, they began to learn to trust more to the curative effects of these resources, collecting together with great industry and zeal numerous observations which served to give support to the *new medical doctrine*, in its application to the study and treatment of surgical diseases. They were less desirous to innovate than to make perfect, and less ambitious of operative success, than of fortunate cures obtained without the infliction of pain or the effusion of blood. The greater the progress that was made, the more confidently was it hoped that the number of cases, requiring the use of the knife and the cautery, might be diminished; and we may further add that, wherever either of these painful methods was still considered inevitable, every endeavour was made to abate the induced sufferings as much as possible, by every thing that professional dexterity and moral influence could suggest

"The art of Surgery thus acquired a character of prudence and security, which were not at all irreconcileable with boldness and decision; and the change thereby effected gave rise to a series of the most pleasing results, by which science, as well as humanity, have equally profited.

"How comes it that this period of improvement has had its epoch of retardation and arrest, and has been only of short continuance?—Ask this question of the spirit of the age, so eager after novelties, so constant in its pursuit of the marvellous, and which has so often substituted fiction in the place of actual reality. There is a restless desire, in the present day, among all classes and estates of men, for brilliant and daring doings; and surgeons have not escaped the general contamination of striving to acquire distinction, without waiting for the sanction of time and experience.

"The first blow that was directed against Physiological Surgery, and which unquestionably has checked its progress in advance, was the division of the art into several sections, or special branches, such as Ophthalmology, Orthopædy, &c. &c.—a practice which now begins to be as much decried, as, a few years ago, it was extravagantly commended.

"It was thought necessary, as we have already said, that surgery, in order that it might not appear to follow either a merely routine or a retrograde course, should follow the experimenting spirit of the age, and join in the career of discovery and empiric research. At the time nothing seemed too difficult for it to accomplish. After having tried with a rare success the excision of the cervix uteri, our surgeons conceived the idea of extirpating the entire organ; after having laid open the abdominal cavity in order to find out the seat of a volvulus or internal strangulation, of the very existence of which the operator was not always sure, they sought to reduce into a precept of the art this most daring and dangerous undertaking, and also to institute a regular operation for the establishment of an artificial anus over the trajet of the Cæcum or ascending Colon.

"Experience had shewn that the section of the tendo Achillis served to remedy certain deformities of the foot; forthwith, the operation of tenotomy was applied to a vast number of tendons without any discrimination, and ere long we heard of surgeons performing it with the view of straightening ankylosed joints, and redressing incurvations of the spinal column!

"Cataract patients were tormented with a variety of operations, in the hope of some plan or other being discovered to extract or depress the crystalline lens a little better than *Daviel,* and a shade worse than *Scarpa.*

"The operation for harelip was the only restoration of the face that used to be practised; but, of recent years, attempts have been made to restore the nose and the eyes, the cheeks and the chin; and, without having much regard either to the amount of pain inflicted at the time or to the troublesome consequences that may follow, the surgeon now-a-days very generally acts with an equal boldness, and often too with nearly the same means, in attempting the cure of a simple deformity, as if the very life of his patient depended on the result of the operation.

"*Hunter* proposed the application of the potential caustic for the cure of certain strictures of the urethra. In course of time, some practitioners sought to make of this method a general plan of treatment, and hence it came to pass that all cases without exception were treated either with caustic, or with incision and scarifications, and the more safe and rational method of dilatation was for a time almost entirely abandoned.

"The school of *Dessault* had much exaggerated the advantage of complicated machines for the general treatment of Fractures, and that of the present day seeks to replace every sort of bandage and splint by using moulds of plaster, in which the fractured limb is to be inclosed.

Really one can scarcely predict where the inventive spirit of our operators might stop, if this state of things were likely to last long without being checked. But fortunately for the interests of our science, these exaggerated notions and opinions, so little accordant with the genuine spirit of enlightened Surgery, are not shared by all. But the voice of reason and truth is for a time drowned in the eager clamour of the moment. Whenever any opposition or disbelief is intimated, we are at once silenced by the sacramental words, *these are facts acquired to science.* But may we not very fairly reply to this seemingly *set-down* argument, that the multiplicity of trephinings for injuries of the skull, the radical cure of herniæ by ligature, the operation for the removal of cataract by keratonyxis, the amputation of a limb to remedy the deformity of a projecting stump, the extirpation of goitre swellings, the excision of varicoceles,—not to mention many other similar undertakings—were, each and all of them, considered at one time to be *facts acquired to science;* and yet where are these *facts* now? are they even so much as ever heard of?

"Having said so much in the way of censure, it might be fairly expected that we should now notice some of the acknowledged improvements and established discoveries, which have unquestionably been made in various departments of surgery since the beginning of the present century. But this we must reserve for another occasion. Enough, at present. As we have already said, it is not that our *science* has retrograded; it is only that our *art* has often exaggerated its importance, and unnecessarily multiplied the cases for its intervention."—*L'Experience.*

---

### A New Method to Promote Cicatrization of Recent Wounds.

The plan recommended by M. *Reveillé-Parise* is not novel certainly; but he has managed to give it an air of originality, by the observations with which he accompanies its exposition. It is nothing more nor less than the careful *Suction* of recent wounds, either with the mouth or by means of an air-pump, as soon after the infliction of the injury as possible. The reasoning of our author, as to the *modus operandi* of this rather antique practice, is ingenious and deserves notice.

"If all wounds," says he, "have a general tendency to suppurate, and if their immediate union is a phenomenon of so rare, as to be almost of exceptional, occurrence, the cause of this is very generally to be sought for in the presence of foreign matters in the wound, which must be discharged, somehow or other, before the process of cicatrisation can effectually take place. It is often very erroneously supposed that those foreign substances are always introduced from without, that they are on all occasions accessible to the senses, and that they may be removed with more or less difficulty, according to the peculiar circumstances of each case, by the hand of the surgeon. Now, so far from all this being the case, they often consist either of the effused or infiltrated blood, of an infinite number of minute coagula plugging up the divided vessels, or of the debris of the skin, cellular tissue, and lacerated or contused muscles, &c.—in very variable proportions, according to the nature of the wound, and the kind of instrument with with which it was inflicted. Contused wounds, as might be expected, exhibit the phenomena now mentioned in the most conspicuous and decided manner. But even in the case of incised and punctured wounds, we may assert that very generally the minute foreign substances, of which we have spoken, are present in greater or less quantity, and interfere more or less with the process of cicatrisation. These organic debris form a *detritus*, the elimination of which must often be effected by the process of suppuration, before the re-union of the wound can take place. To cleanse a traumatic surface is therefore a primary and most necessary indication, if we expect to induce its immediate cicatrisation, or even to abridge the time necessary for this process being effected. To effect this important object, no method is at once so simple and effectual as that of immediate Suction."—*Bulletin de Therapeutique.*

---

### M. CAZENAVE ON THE TREATMENT OF PSORIASIS.

Of local applications, two of the very best are certainly the ointments of the Ioduret of Sulphur (one or two parts to 30 of lard), and of Pitch (one part to eight of lard.) M. *Biett* (the original proposer) and M. *Cazenave* have obtained most satisfactory results from the use of the former; but then let it be remembered that internal medication was diligently pursued at the same time. There cannot however be a reasonable doubt, but that the Ioduret of Sulphur exerts a salutary modifying action on the dry and squamous state of the skin in such diseases as Lepra and Psoriasis.

M. *Cazenave* has, on more occasions than one, had an opportunity to observe that the application of the ointment by friction to one part only of the affected surface—for example, to one extremity—has been followed not only by a cure of the disease there, but also by a decided amendment of it in other parts of the body. The Pitch ointment should be applied to the scaly patches twice daily. The occasional use of an alkaline or of a vapour-bath will much promote the celerity, as well as the certainty, of a cure. The great objection to the use of the Pitch application is its smell and nastiness; bating these, it is certainly a most useful remedy. A variety of different sorts of Baths have been recommended in the treatment of squamous diseases; and certainly the practice of general ablution, in some form or another, will never be omitted by the judicious physician.

A very simple and serviceable alkaline bath may be prepared by dissolving the common carbonate of Potash in the water—in the proportion of from four to eight parts of the salt to 500 parts of the fluid, or of from four to eight ounces to an ordinary tepid bath. The sulphur-baths may often be used in obstinate cases with very decided advantage. Neither M. *Biett* nor M. *Cazenave* have ever witnessed any good effects from baths containing sublimate of mercury, as recommended of late years by several writers. But of all baths, according to their experience, the simple water-vapour bath is unquestionably the best. As a general remark, the use of this excellent remedy should never be neglected; and no other sort of bath need ever be had recourse to, until a fair trial has been made of the steam one.

As to internal remedies, the catalogue of those which, at different times, and by different writers, have been recommended, embraces a vast number of the substances in the Pharmacopœia, besides many extrinsic and more homely formulæ. The most approved are unquestionably the preparations of sulphur, of antimony, and of arsenic, and the tincture of lytta. Sulphur, in various forms, is well adapted to young patients and persons whose skin is fair and also irritable, more especially when the disease is comparatively mild; but in inveterate cases of the disease it generally fails. M. *Cazenave* speaks highly of the Plummer pill as a most useful antimonial: sarsaparilla tea, to which a little mezereon has been added, may be taken with advantage at the same time. He has not often made use of the tincture of cantharides; although he acknowledges that, on many occasions, he has seen remarkable benefit derived from its administration, in the practice of M. *Biett*. But of all remedies, none are, in his estimation, equal to the preparations of arsenic. Under their use, the diseased patches, sooner or later, become warm, red, and often somewhat tumefied; at the same time the scales drop off from their surface, and they exhibit at first an erysipelatous appearance, which is subsequently replaced by a pale hue of the skin—a precursory symptom of the shrinking and progressive disappearance of the patches. In the *Lepra Vulgaris* the disks become partially broken up, their edges subside, the circles lose their rounded figure, and the debris of each ring proceed to resolution, often with a truly surprising rapidity. In our author's opinion, arsenic and the simultaneous use of vapour-baths, constitute *the* treatment of Psoriasis and Lepra: occasionally the application of some pommades, at the same time, may accelerate the cure.—*Annales des Maladies de la Peau.*

*Remarks.*—A much safer, and often quite as efficacious, a remedy for Psoriasis as Arsenic, is the Liquor Potassæ, given in doses of from 15 to 30, or even 50 drops, thrice a day, in milk or beer. A weak solution of nitric acid in water will be found, in many cases, an excellent application to the scaly skin. As a matter of course, the use of baths should never, if possible, be omitted. If a regular bath cannot be procured, the free ablution of the entire surface with tepid water should be practised every night, before going to bed.—*Rev.*

---

### Strychnine a Test for the Administration of Morphia in Neuralgia.

Dr. *Rougier*, one of the physicians of the Hôtel Dieu at Lyons, published, last year, a work—entitled, "De la Morphine Administrée par la Methode Endermique dans quelques Affections Nerveuses, et de la Necessité de l'Usage interieur de la Strychnine pour achever le traitement et prevenir la recidive, &c."—in which he has endeavoured to shew that the latter alkaloid may be used as a *test* to try the efficacy and permanence of the sedative effects of the former, in certain cases of painful disease. The Morphine is administered in the Endermic Method; and, for this purpose, Dr. R. prefers the use of the heated hammer (as recommended by M. *Mayor* of Geneva) for the purpose of vesicating the skin, to that of an ordinary blister, or of the *pommade ammoniacale*. From half a grain to five grains or even more of the morphine may be sprinkled on the raw surface. By this powerful means, the neuralgic pain is usually speedily lulled, if it be not thoroughly and effectually dissipated. Dr. *Rougier* acknowledges that there is a greater tendency to the relapse of the pain after the endermic, than after the internal, administration of the remedy. With the view of determining whether the relief, once obtained, is likely to be permanent, he says that he has found the exhibition of strychnine to constitute an almost unerring *test*. The manner in which he employs it is the following. He begins with one-eighth of a grain twice a day, and gradually raises the dose until a grain or a grain and a half be taken in the course of the 24 hours. When, after a few days' use of the remedy, the twitches, which it generally induces in the affected limb, become so severe as to re-produce the neuralgic suffering, we may infer that the cure has been complete, if the pain daily becomes less and less, in spite of the increase of the doses. In such a case the twitches usually continue, increase in frequency and intensity for a time, but ultimately become rather inconvenient than positively painful. The use of the strychnine, as a re-agent or test of the efficacy of morphine, may then be suspended; unless indeed it be deemed advisable to continue it for the purpose of giving tone to the enfeebled muscular tissues. In the second case, on the contrary, the pain always goes on increasing, and forces us to abandon the exhibition of the remedy: the Neuralgia, having become as severe as it was before any morphia had been employed, shews that the Endermic Method had not been carried to a sufficient extent. Under such circumstances, Dr. *Rougier* recommends that it should be resumed. "An important remark," adds he, "that we have made is, that the disease, when resuscitated by the use of the Strychnine, is usually much more acute and rapid in its course than before; also, that the saturation of the system by the opium is then much more readily obtained; and that the strychnine, employed anew, has never failed to insure the patient against the eventuality of a second relapse."

Dr. *Rougier*, at the close of his work, relates several cases to prove the efficacy of Strychnine in obstinate cases of Chorea. His experience of it in this disease preceded by three or four years that of M. *Trousseau*, who has of late lauded the remedy so highly. Dr. R. acknowledges that he derived the hint from *Bayle's* Bibliotheque Therapeutique, (published in 1830,) where we read: "M. *Cazenave* has had recourse, with decided success, to this medicine (nux vomica) in a case of St. Vitus's Dance, which had resisted the use of all the most approved remedies."—*Gazette Medicale.*

---

### UTILITY OF SAVINE IN ATONIC METRORRHAGIA.

After some severe, but well-deserved, remarks in censure of the utter neglect of general Therapeutic Medicine during the reign of the self-called physiological school, Dr. *Aran* hails the return of a spirit of more unprejudiced investigation touching the curative virtues of many remedies, that were highly esteemed by our forefathers. Of these Savine, as an Emmenagogue, is one that deserves the notice of every practical physician. In reference to the *modus operandi* of this class of medicines, he very justly remarks: "We can readily conceive that, at a period when the Catamenial Secretion was regarded as merely a sanguineous congestion of the uterus, followed by a discharge, and occurring without any apparent use or object, medical men might seek to induce mechanically a stasis of blood in the cavity of the uterus, although doubtless they would have succeeded better if recourse had been had to drawing blood from the feet, and the employment of hot hip-baths, &c. But, since the interesting researches of MM. *Gendrin* and *Negrier* have so satisfactorily proved that the menstrual exhalation is not an effect without a cause, and that it is directly and immediately connected with the passage of an ovule from the ovarium into the uterus, we can easily understand that the design of Nature would not be at all promoted, if we limited our efforts, in cases of amenorrhœa, to the mere induction of uterine plethora. The evolution of this ovule is a normal act in the adult female, and, like all the important efforts of the *nisus formativus,* it must require for its fulfilment the regular and healthy exercise of all the functions of the body. Hence it is to this end that all the aims of the physician should be directed, and certainly not to merely determining an accumulation of blood in the substance of the womb—a state of things, moreover, that is not always free of danger."

"Although the Emmenagogue properties of Savine were well known to many of the ancient physicians, it does not appear that its power in arresting uterine hæmorrhage was recognised, until *Wedekind* (Hufeland's Journal) in 1799 wrote a valuable memoir on the subject. He employed it extensively not only in Atonic Metrorrhagia, but also in numous cases of Leucorrhœa, with the most satisfactory results. But its reputation does not seem to have lasted long; and it had fallen into entire neglect, when *Gunther* (Hufeland's Journal, 1826) and *Sauter* (Melanges de Chirurgie Etrangere) again brought it into notice. The latter writes thus of its virtues: "Savine is one of the most powerful and valuable remedies, not only against sanguineous discharges and other diseases that are independent of the existence of pregnancy, and are characterised by the names of atony, asthenia, deficient contractility or power of cohesion, but also against the hæmorrhages which often precede and make us fear the occurrence of abortion, especially in women of a lax fibre who have already miscarried. I have administered the Savine in doses of from 15 to 20 grains of the powder, three times a day, with very decided success, in numerous cases of uterine hæmorrhage and threatened abortion."

The remedy has been tried pretty extensively in M. *Gendrin's* practice at the Hôtel Dieu, and with very marked success, in numerous cases of uterine hæmorrhage, whether this occurred independently of gestation, or after delivery. The remedy is best suited to those cases, where there is great laxity of the system, and the hæmorrhage seems to be owing to loss of power in the blood-vessels of the womb. M. *Gendrin* occasionally combines it with sulphate of iron.—*Bulletin de Therapeutique.*

*Remarks.*—From this notice, it is sufficiently obvious that the properties of Savine are very like those possessed by the Ergot of rye. Both seem to exercise a direct stimulant effect on the muscular tissue of the uterus; and hence both have been employed on the one hand as emmenagogues, and on the other to arrest atonic hæmorrhage from this organ. We need scarcely add that no judicious practitioner will administer either remedy in plethoric states of the system, or whenever there is any tendency to inflammatory excitement.—*Rev.*

---

### Remarks on Iliac Phlebitis in Phthisical and other Patients.

The Statistical Tables published by Dr. Burns satisfactorily establish the fact that the disease of *Phlegmasia alba dolens* occurs much more frequently (nearly nineteen times out of twenty) on the left than on the right side. Can we give any reasonable interpretation of this circumstance? May we not suppose that the passage of the blood along the left iliac vein is apt to be more or less seriously obstructed, in consequence of the not unfrequent accumulation of hardened fæces in the sigmoid flexure of the colon?

In certain states of the system, and especially in that which has been called the Purulent Diathesis, there seems to be a tendency to some of the internal veins of the body becoming inflamed, and to the consequent coagulation and deposition of the blood within their cavities. It has been conjectured by some pathologists that this change of the blood precedes, and indeed is the cause of, the adhesive inflammation of the vascular parietes; and this may, very probably, be the case when pus or any other poisonous matter becomes mixed with the circulating fluid. Whatever view we take of the subject, there is no doubt that the occurrence of Phlebitis of the iliac or femoral vein, during the course of any protracted disease, is always to be regarded as a very unfavourable circumstance; seeing that a cure very rarely takes place when the disease is once fairly established. Phlebitis of the iliac vein is of not unfrequent occurrence in phthisical patients. In one of the cases related by Dr. *Toulmouche*, we read "that the left leg became very œdematous, the right one remaining quite unaffected. This swelling, as well as the existence of pain and tumefaction on the left side of the abdomen, and of tenderness along the course of the veins of the affected leg, made me suspect that the iliac vein on this side had become inflamed, and its tube partially obstructed by coagulated blood. The œdematous swelling became greater and greater, while the wasting of the rest of the body gradually increased. For two months before death, a distinct fluctuation of fluid within the abdomen might be perceived. * * * * * *Dissection.*—Not to mention the changes of the internal viscera, we proceed at once to notice the condition of the iliac veins. The right one was found to be perfectly normal; but the left, immediately below the bifurcation of the inferior cava, was filled with coagulated blood; and its parietes, along with the adjacent cellular tissue, were thickened. On exposing the vein and its divisions along the entire length of the limb, I observed that the iliac was much hypertrophied, and that its canal was plugged up with blood, which was the more distinctly fibrinous and the less deeply coloured, in proportion as the vessel was examined the nearer to its point of origin; whereas, in the femoral portion, the blood, though quite concreted, was dark and not so firm and consistent. The collateral branches were similarly affected. Every thing led me to believe that the phlebitis dated from the period, at which the patient began to experience pain in the deep-seated part of the abdomen on the left side, and in the corresponding extremity. When the vein was cut across, its parietes were found to be actually thicker than those of its fellow artery. The thickening of the venous tube fairly extended down below the ham." It may be mentioned that in this case there had been, at one period of the disease, the symptoms of Pericarditis—sharp pains in the region of the heart, accompanied with palpitations—and that, on dissection, a considerable quantity of serum was found in the pericardium.

In another case, where a patient died of Necrosis of the 11th and 12th ribs, accompanied with tuberculous disease of the lungs, the left lower extremity became enormously swollen with œdema, three months before the period of dissolution, while the rest of the body was frightfully emaciated. "On dissection, the left iliac vein exhibited the following appearances. Immediately below the bifurcation of the inferior cava, it (the iliac), at first almost quite empty, was found to be gradually filled with a fibrinous concretion, which had the effect of dilating it and making it appear firm and distended, as if it had been injected along the rest of its course, and so that the insulation of it and of its branches by dissection was rendered very easy. On laying it open longitudinally, it was observed to be filled, as far down as the middle of the thigh, with a coagulum adhering very strongly to its internal tunic, which was red in some points, black in others, and considerably thickened in substance. The parietes of the vessel, when cut across, were as thick as those of the aorta. The fibrinous coagulum terminated in a fusiform manner,—upwards to near the origin of the vein, and downwards to a little above the third adductor muscle of the thigh. The Saphæna vein also was much thickened, as well as several of its ramifications. The intermuscular tissue of the thigh was exceedingly infiltrated, and cut, under the knife, like bacon. The muscles themselves were pale and exsanguine."

In this, as in the preceding case, the invasion of the Iliac Phlebitis was announced by the supervention of œdematous swelling of the left lower extremity, the right one being not similarly affected, but extremely emaciated.—*Gazette Medicale.*

---

### Notice of some recent French Works on Typhoid Fever.

"The interval, that separates Continued from Intermittent Fevers, is far from being the same in all cases and in all countries. There are localities where these states of Pyrexial disease,—habitually so distinct in our temperate climates—touch and almost blend with each other, so that it is difficult to say to which class individual cases, or even groups of cases, are to be referred.

"We do not at present allude to those purely hypothetical approximations that exist only in the imagination of certain authors; for example, the opinion of some Pyretologists, who seek to attribute all fevers, without exception, to the agency of Miasmatic poison, whether this arises from a vegetable or from an animal source. The approximations which we mean, are real and complete; yet such as may baffle and perplex the most experienced physicians, and in consequence of which the patient may fall a victim to an almost inevitable error of judgment. These ideas, now so generally recognised and adopted, were scarcely so much as dreamed of a few years ago,—at the period when medical researches were confined within narrow trammels, and a spirit of scepticism in reference to everything that could not be *seen*, and *felt*, and *handled*, prevailed far too extensively among the schools. But since medicine has begun to be enriched with the observations of men of almost every country of the world, and especially since distant expeditions have enabled *French* physicians to put to the test of actual experience the narrow rules and instructions which they had derived during their professional pupilage, our science has made rapid strides in advance, and the utter insufficiency of the study, however elaborate, of fevers as observed in any one climate, is now very generally recognised. At the same time, several learned writers have, from the stores of their erudition, taught us the admonitory rebuke that, on this as well as several other themes, Modern Science has forgotten and neglected many of the lessons of Ancient Medicine: of these a very important one is, that the same morbid condition may exhibit different forms and aspects in different climates and localities, as well as in different seasons and periods of the year.

"We cannot do better, in confirmation of the truth of these remarks, than quote the following passage from the work of Dr. *Gouraud*, 'On the Pernicious Intermittent Fever of Southern Climates,' published two years ago, at Avignon. 'The medical men,' says this gentleman, 'of the North, if at a leap they wish to practise in the South, fancy that grave periodic fever exhibits the same march and a like physiognomy everywhere; that it differs very little from what we see in the fevers of our own country; and that every accession or paroxysm has three stages, with distinct intermissions between each attack. They are not prepared to expect that eschars should form on any part of the surface, except where a certain degree of pressure is kept up; nor can they believe that the occurrence of *psora labialis* should fail to be regarded as invariably a most favourable symptom. Accordingly, they take their own time, bleed or vomit their patients, according as either the pulse is full or there is any sign of hepatic congestion, and fear much less to practise an ill-timed blood-letting, than to administer quinine before the febrile symptoms have fairly subsided. In short, these new practitioners of the Morea (we suppose that the author is alluding to some of his professional brethren in Greece,) are quite as 'depaysés' at the bedside of their patients, as if they had passed from reading the first and third books of *Hippocrates* on Epidemics, to the perusal of the writings of M.

*Andral.* Such a temporisation too often proves fatal to the patient; and for this simple reason, because the Summer periodic fevers of warm climates are separated from those of northern latitudes by a broad abyss.'

"Dr. *Gouraud* censures very sharply the conduct of the medical officers of the French army in Algeria, for practising bloodletting so generally as they do at the commencement of the grave remittent fevers of that country, and for their timidity in administering quinine and other tonics.

"In the course of last year, Dr. *Waton* of Montpelier published a treatise on the Typhoid Fever, as observed in that city, and in which he powerfully recommends the exhibition of quinine, not as a tonic or contra-stimulant, as some have proposed, but chiefly as an anti-periodic. 'We shall not be surprised,' says he, 'if the recent experiments, which have been made in the hospitals of the metropolis on the use of Quinine in Typhoid Fever, have not produced so advantageous results as might fairly have been expected from them, when we learn that, the disease being not regarded as essentially remittent in its nature, the remedy was administered quite indiscriminately, and without any regard to the existing condition of the patients; moreover, that the exhibition of it was often not preceded by the use of any precursory means, the adoption of which is sometimes absolutely necessary, in order that the Quinine might produce its accustomed effects; and lastly, that it was given in a multitude of complicated cases, where it could not reasonably have been expected to have produced any benefit.'

"According to the opinion of Dr. *Waton*, Typhoid Fever is, at least in many cases, a remittent affection—characterised, not indeed by the occurrence of distinct and complete intermissions, but by a continued pyrexia which, however, exhibits regular exacerbations. These exacerbations occurring twice in the course of 24 hours, are sometimes so indistinctly marked as not to be readily recognised. If not intense, they cease under the employment of an antiphlogistic medication; but, if they are of a more grave character, they yield only to the prompt and decided use of Quinine. Whenever the exacerbations are not severe, and are complicated with any symptoms of inflammatory or congestive action in an internal organ, Dr. *Waton* recommends that blood should be drawn either locally or generally; for it is not, until after this has been done, that the Quinine acts energetically in arresting the progress of the fever under such circumstances.

"The quantity of this salt, that requires to be administered, varies from 10 to 100 grains in the course of the 24 hours, according to the nature of the individual case.

"Dr. *Waton*, in the course of his observations, very freely criticises the practice that was adopted in several cases, narrated in the works of *Andral, Louis, Chomel* and others, and in which there had been more or less distinct exacerbations of the pyrexial symptoms during the day. In reference to one of M. *Andral's*, he thus remarks:

'I may conscientiously assert that it is my deep conviction that, if the quinine had been freely administered in this case, the physician might probably have saved the life of his patient.'

"Dr. *Waton* mentions an important circumstance that may not be generally known; it is this: that, if the exacerbations are distinctly marked, even although symptoms of inflammatory action be present at the same time, the quinine may be administered with perfect safety, provided only that antiphlogistic measures are simultaneously adopted.

"Connected with the history of Typhoid fever, we may here allude to another work on the subject that has been recently published by Professor *Rangue* of Orleans. The gist of its contents is contained in the following extract: 'There are certain symptoms by the aid of which we may predict, from the very commencement of a fever, its character and course. During an experience of 18

years, we have never found them fail, as a most valuable means of guiding our prognosis. Whenever, during the early days of a continued Fever, whatever be its form or type, there is deposited on the gums, interposed between the molar teeth, a white pearly-looking exsudation, we may feel assured that the disease will assume a grave character, if it be not treated judiciously. This symptom is of constant occurrence, not only in the Pyrexiæ, but also in all affections that are apt to become of a Typhoid character, such as puerperal fever, pneumonia, &c. If to this symptom be super-added the mulberry-juice tint of leech-bites, and commencing prostration of strength, the affection has from this moment the Typhoid character, however favourable the general symptoms may seem to be at the time. * * * To assure ourselves of the state of the gums, it is requisite that the attention should be specially directed to the part of the gingival surface, that invests the small molares. * * * The surface of the interstices, which separate these teeth, exhibit a nacreous pyramid, the base of which rests on the body of the gum. * * * If the exsudation, which forms this nacreous layer, is very thin, and occupies a small extent, the disease will be 'peu grave;' but, if it be thick and not easily removed with the finger, if it affects a number of the dental interstices, and it is of a grayish colour, we may confidently predict the gravity, if not the malignancy, of the disease. * * * * * In certain cases, the nacreous exsudation is not limited to the gums, but extends over a larger or smaller surface of the buccal and pharyngeal mucous membrane, and it then constitutes one of the most grave symptoms of typhoid affection."

*Remarks.*—It is surely very fanciful to attach much importance to so subordinate a symptom as that of the gingival incrustation, as a sign to guide our prognosis in Typhoid Fever. Is not the state of the pulse and of the nervous power, not to mention that of the excretions, abundantly sufficient for this purpose? The practical observations of Drs. *Gouraud* and *Waton* are much more deserving of notice; and we sincerely trust that the advice of these gentlemen will tend to promote a more rational system of treatment of Pyrexial disease than has, for many years past, prevailed among French practitioners. We verily believe that the doctrines of *Broussais,* and his disciples, have done more to distort and pervert medical truth than all the fond vagaries that prevailed from the time of *Paracelsus,* down to that of *Brown* and his followers in Italy.

---

### Moral Theology, in reference to Physiology and Medicine.

Such is the title of a work, published last year in Paris, by M. *Debreyne,* doctor of medicine, and priest and religious brother of the order of Grand Trappe. It is intended chiefly for the use of the clergy, as a guide-book to instruct them upon many topics that appertain conjointly and simultaneously to the welfare of the soul and the body. A great variety of subjects is introduced and discussed with excellent judgment—according, at least, to the opinion of the reviewer in the Revue Medicale, to the pages of which journal we are indebted for our knowledge of its contents. Our readers, we think will be pleased to learn something of a work, which, while in many parts it breathes a fine spirit of religious feeling, seems to be tinctured with not a little of the amusing extravagance and ignorant bigotry of the old schoolmen of the Sorbonne.

M. *Debreyne,* as might be supposed, is a great admirer of the olden school of Physiology, as the science existed in and about the time of *Haller.* He insists much on the importance of attending to the differences of Temperament in different individuals—retaining the old quaternary division into the bilious, the sanguineous, the lymphatic and the nervous.

On the physiological characters and the pathological tendencies of the last-named constitution of the body, he dwells at great length, and points out the evils, physical as well as moral, that it is most likely to give birth to. The many perturbations of social life, the immense extension of unbridled luxury among all classes, the general corruption of moral principles, the melancholy absence of proper religious feelings, the licentious, romantic (as it is called,) style of modern literature—these are the chief causes of the predominance in the present day of the Nervous Temperament, which is, it should be well remembered, less a natural constitution than a pathological condition of the system. M. *Debreyne* shews, with great earnestness, the paramount importance of educating the *heart* as well as the *mind* of young people, who are so endowed. "If the imagination," says he, "comes once to prevail, and the judgment is not rightly formed at the same time, the character of the individual will become intractable, proud, and insincere, with a sensibility that is apt to be excessive, and to be perverted by the imperious domination of violent passions. In vain may we hope to subdue this moral insanity, by the pompous dogmas of mere human philosophy;—empty labour, fruitless endeavour! Such a state of alienation of the heart can only be cured by the therapeutics of religious principles, and the power of a Christian education:"—a truth that every one is ready to acknowledge in conversation, but which is, nevertheless, almost always forgotten in the education of our children; although, to use the beautiful language of our author, "*there* lies the help and medicine for all deeply-suffering souls, and, apart from it, there is no future peace, nor life, nor safety for them, either in this world or in the world that is to come."

After giving a most faithful description of what he calls the "erotic temperament," and exposing the many ills and dangers to which it so often leads its victims, M. *Debreyne* treats, with a rare sagacity, of the offensive subjects of personal pollution and conjugal Onanism. This part of the work is written principally for the direction of Priests in the solution of some delicate questions, which often come under their notice in the secrets of the Confessional.

The third part has this curious heading—of *Sacred Embryology*. In reference to the nice point when the fœtus may be considered as quick, or endowed with life, our author holds the orthodox doctrine, as expounded by *St. Basil* and *Cangiamila*, that animation dates from the very moment of actual conception,

"If," says he, "the life of man ceases immediately upon the separation of the soul from the body, we may with equal reason believe that the soul becomes at once united with the body, however minute and rudimentary the latter may be. Now, as soon as the ovule is fecundated—an act which takes place at the very moment of consummated generation—it has Faith, or, in other words, it believes; believing only because it is alive, and being alive, only because it is animated. The human germ or ovum is therefore animated at the very moment of impregnation.

"Moreover, do we not know, that the soul remains united with the body up to the very last moment of the mortal agony, even when almost every organ is already smitten with paralysis and death? and can we suppose that this weak breath, this feeble spark of material life, which is trembling on the very eve of extinction, possesses a higher vitality than the life of the fecundated embryo, which, all must acknowledge, has at least many formative, plastic, and increasing energies within it?

"Let not the physical minuteness of the being surprise our weak reason! God is always great and infinite alike in small and in large things; or, we should rather say, there is nothing in the universe either large or small in His sight. The relative qualities of size are but the creation of our feeble understandings, and the enunciation of which is only necessary here below to enable us to appreciate the order and harmony of the material world." Our author having thus most satisfactorily (!) proved that the Fœtus is a living being, endowed with a soul from the very moment of conception, we must be prepared to admit—(with Uncle Toby)—that every abortion, however premature, provided only that it exhibits signs of life, should be *baptized.* * * *

" Moreover, as the uterine life of the Fœtus is not immediately dependent on that of the mother, it is obviously our bounden duty, whenever a pregnant woman dies, to extract, as quickly as possible, the child from her womb, in order that it may receive the holy sacrament of Baptism, if it be yet alive. For, in spite of many assertions to the contrary, there is good reason to believe that fœtal life often continues for a considerable time after the death of the parent. *Millot* tells us of a case, where the fœtus was found alive no less than 48 hours after the mother's death!"

M. *Debreyne* has triumphantly refuted the opinions of Professors *Velpeau* and *Moreau,* who deny the truth of these long survivals, question the accuracy of the facts recorded by *Cangiamila,* and recommend that, in such circumstances as we have alluded to, the fœtus should not be extracted from the uterus, unless it is at least five months old;—as if forsooth its spiritual attributes were to be measured by the number of weeks of its life! But, thanks to our pious author, we may now entertain the hope that a certain number of fœtuses, who might otherwise have perished, shall be saved, if not for time, at least for eternity!!

With respect to the much disputed point of medical duty, whether, in cases of difficult labour, we are ever justified in sacrificing the life of the child with the view of saving that of the mother, M. *Debreyne,* after much deliberation, determines it in the negative. " There is," says he, " a maxim that is of infallible and everlasting truth, *non sunt facienda ut eveniant bona.* The precepts of the Natural Law never admit of any dispensation whatever; and, therefore, under no conceivable circumstances, can we ever be allowed to take the life of an innocent being. But you will perhaps say, that, if the fœtus is not destroyed, the life both of it and of the mother will almost inevitably be lost. Doubtless, this is a deplorable misfortune; but, remember, by sacrificing the child, you are not sure of saving the mother; for we are expressly told by M. *Giraud* (Journal de Medicine, par *Corvisart* et *Boyer,*) that he has often witnessed the operation of embryotomy performed, and that the mothers have generally died immediately afterwards. Besides, we should never forget that, although the child may not survive for even an hour after its birth, it may yet have received the greatest and most precious of all blessings, that of Baptism."

In the *fourth* chapter, M. *Debreyne* treats of Animal Magnetism, &c. (We may here mention that Phrenology receives no favour at the hands of our reverend author: he condemns it as utterly groundless and contradictory). The good old man shrewdly suspects that not a few of the results, produced by this marvel-working agent, are due to the mere excitement of sexual passion. " Suppose," says he, " (and indeed this is almost always the case) it is a woman that is to be magnetised, and that it is a man who is the operator; what is the very natural consequence of the 'rapports' established between them? The imagination of the former, concentrated on what is expected to take place, and sensitively alive to the lightest pulsation and to every organic movement of her body, is apt, in highly impressionable constitutions, to experience a thousand unknown sensations;—not indeed that these arise from the agency of any magnetic influence, but merely from the attention of the individual being riveted, probably for the first time, on certain corporeal movements which were always existent, although perhaps never felt or experienced before. And, as it is a man who performs the operation, and as a woman's imagination feels, so to speak, his presence with all that increased exaltation of sensibility that has come upon her, it is the uterine system that experiences the impressions more strongly than any other part of her body. Movements, at first of a confused and gradually of a more distinct and intelligible nature, arise in these organs; and feelings, of a vague sensuality in their commencement and subsequently of a more lively and warm desire, are produced. If the woman be chaste, such a state agitates and exhausts her system; if otherwise, it excites violent passions which are made abundantly obvious by various erotic movements. Can we wonder at all this, when we know that the magnetiser—independently of making the *passes* and of fixing his eye upon her—often takes her hands between his, and then draws his fingers over various parts of her body, now over her face, and then over her body and legs, pressing perhaps his knees against her's, and sometimes applying (we have seen this done) his lips to her stomach and making insufflations upon it. Have we said enough to shew that—in some cases, at least—the use of Animal Magnetism is morally dangerous? We have heard it acknowledged by a most zealous practitioner of the art that he has, more than once, witnessed all the excitement of actual coition thus produced in a woman. We cannot therefore wonder that Animal Magnetism, as it is called, should give rise to certain energetic effects in very impressionable systems; and, although we cannot for a moment admit the credibility of a tithe of the marvels we have heard of, yet we are not prepared to deny that it is capable of inducing some rather remarkable phenomena. Of these, one of the most constant is the Magnetic Sleep, into which certain patients are easily thrown, when operated upon. This state is not to be regarded as a normal or physiological, a natural and refreshing repose; it is rather an abnormal and unhealthy stupor, that is apt to be induced by certain 'rapports' that are more or less fitted to make a strong impression on the nervous system of highly excitable persons. This stupor does not differ in its nature from that which spontaneously occurs in some persons during ordinary slumber. In both cases it is a genuine neurosis, which not unfrequently exhibits certain so-called phenomena of *lucidity*, and during which the person is able to do what he could not in a state of waking. Now, as this lucidity is observed in certain lesions of the encephalon, and occasionally too in hysteria and catalepsy, it comes within the domain of the physiology, or rather of the pathology, of the nervous system."*

M. *Debreyne* devotes a chapter to the consideration of reputed Miraculous Cures, treating the subject with singular moderation and sagacity. Unwilling to recognise the intervention of any supernatural agency in most, if in any, of the alleged cases, he regards them as nothing more than affections of the nervous system, genuine Neuroses, that are often kept up or even aggravated by the means used to relieve them. Our worthy author relates the history of a curious case, in which it was alleged that a young woman, besides having *stigmata* on her bosom and feet, was in the habit of receiving bits of sugar and even roasted apples in a miraculous manner. The case was submitted to his judgment, to determine if any credit was to be attached to the story. His reply is so good that we must give one or two extracts from it. * * * * "This extraordinary phenomenon must be either a divine miracle, or a false one performed by the Devil, or lastly it may be a mere human trick and deception. 1. It cannot be the first; for we can discover in it no end or purpose, worthy of the interposition of

---

* Animal magnetism—and, by the bye, Phrenology also—has now become quite a popular and attractive amusement. We observe by the bills of the Adelaide Gallery that, three times a week, there is "a lecture on Mesmerism, accompanied with experiments to shew the phenomena of Clair-voyance, Mental Transfer, Phreno-Magnetism, Community of Taste and Feeling, Lucid Somnambulism, Catalepsy, and Mesmeric Coma!" Among the other attractions, there is a Phrenological Exhibition! the Steam Gun! Electrical Eels! a Musical Voyage! and a Pictorial Concert!

divine agency. It obviously does not serve to advance either the glory of God, nor the good of the Church, nor the salvation of souls, nor yet the welfare of humanity: in a word, this pretended miracle displays no feature of resemblance with those recorded in the Holy Scriptures; it has produced no result that is praiseworthy, edifying, or useful to mankind. We therefore are convinced that it does not proceed from God: *Digitus Dei non hic est.*

"2. It is not the work of the Devil; we cannot see what interest he can have in it. Unhappily, the Evil Spirit too easily succeeds in corrupting the minds of young persons, by feeding them with the honey of carnal and unworthy pleasures; and certainly he has no need for this end to make them eat *sugar* and *roasted apples.* Alas! in the present day, young girls are often depraved from their very cradles, *erraverunt ab utero.* In the corruption of their morals, they lose their souls, and often too, if not their lives, at least their health and comfort. How many young persons, in the morning of their existence, may say with the poet *Gilbert,*

> Au banquet de la vie, infortuné convive,
> J'apparus un jour, et je meurs:

They have tasted the baneful honey of pleasure, and though scarcely entered upon life, they, like flowers that have fallen under the scythe, fade and wither; they die, and are no more—*gustans gustavi paullulum mellis, et ecce ego morior.* In the examination of such questions as the present, we should often recal to our minds the sage remark of Marescot: '*à naturâ multa, plura ficta, à demone nulla.*'"

We need scarcely add that M. *Debreyne* decided that it was all a deception on the part of this girl.

The last chapters of the work treat of Fasting, Abstinence, and such-like matters, not only in a physiological and hygienic, but also in a moral and ethical, point of view. We are then presented with a succinct narrative of the practices of the modern *Trappists,* with remarks on their longevity compared with that of other men in different situations of life.

In concluding his notice of this curious work on Modern Theology, the French reviewer remarks that, "if it produced no other good than that of earnestly inculcating the benefits, as well as the duty, of greater holiness and virtue in all the conditions of life, it deserves to be widely and very generally known.

This advice is applicable, in an especial manner, to all medical men. We have high authority in our favour. Has not *Hippocrates* laid it down as a necessary qualification of a physician, *primum a divinis numinibus auspicetur;* and does not *Avicenna* inculcate a similar precept in the following beautiful words? "Nec ullus medicorum, quamvis artis precepta ad unguem usque perceperit, quicquam magnâ laude dignum, aut cum prospero successu consequi poterit, nisi *pietas* ipsi curæ cordique fuerit?" It has also been forcibly remarked by an old writer: *Sanctitatis autor Deus; Dei instrumentum Natura; utriusque minister medicus est.* The perusal of such a work as this of M. *Debreyne* will, we have every reason to believe, have no inconsiderable influence in diffusing sounder and better views among many medical men than have unfortunately hitherto prevailed. It has too often been a reproach to our profession that so many of its members are, if not infidels, but very little under the influence of religious feelings. Alas! the charge is but too true; but let it not be imagined that it is the study of medicine that corrupts the heart; it is rather the corrupted heart of the medical man that depraves his profession."

---

### On the Cure of Stammering.

Dr. *Becquerel,* having been cured himself by following the plan recommended and practised by M. *Jourdant*, has, with the permission of the latter gentleman, published an account of the mode in which he has been relieved of his distressing infirmity. The readiness with which M. *Jourdant*—although described, in one of the French journals, as an obscure mechanic—granted permission to reveal the nature of his secret, is highly honorable to him, and deserves the thanks of the medical profession.

What is the nature and primary cause of stammering? According to MM. *Rullier, Voisin, Colombat, Bell*, &c., it arises from an inability on the part of the patient to co-ordinate and combine together the various actions of the vocal organs, and especially those of articulation. The 'point de depart' of this lesion of the vocal functions is unquestionably seated in the nervous system. A clever writer has defined it to be a Neurosis, consisting in a choreic spasm of the expiratory muscles, and a tetanic rigidity of those which regulate the production of the voice. We need scarcely say that the idea of there being any actual shortening in the dimensions of the tongue—an idea, by-the-bye, which gave rise to the 'funeste' proposal of cutting the *genio-glossi* muscles for the cure of stammering—is utterly without foundation.

M. *Jourdaut* is of opinion that the cause of Stammering consists in the precipitate emission or escape of the inspired air and of the speech at one and the same time; the stammerer expends in *blowing*, and not in *sound*, the air which he has received into his chest. Let us for a moment consider the series of phenomena, which take place in the natural and healthy condition of the organs. Whenever we wish to produce any vocal sound, we first of all make a deep inspiration and dilate the chest; then, at the moment when the act of expiration commences, the air during its escape through the *rima glottidis* produces the sound desired, and this is continued until all the inspired air has been expended. When this is the case, the person either ceases to speak, or, if he wishes to continue to do so, he makes a new inspiration, and the same succession of phenomena is reproduced and repeated. From the moment when the act of expiration begins, the chest returns to its normal condition slowly and gradually, in proportion as the sound is emitted; and the inspired air is so managed, that it escapes the more slowly as the sound is the more intense and prolonged.

In the case of the stammerer however, no sooner has the air been inspired into the chest, than its parietes begin to contract too soon, in order to expel any excess received. The sudden contraction expels a greater quantity of air than is necessary for the production of speech; and then this large volume of air, coming into the mouth at the moment when the tongue, the lips, and the cheeks are contracting for the purpose of articulating the sound, opposes the regular action of these organs: in this manner the confusion of the speech is induced.

The primary Cause therefore of Stammering consists, if this view of the case be correct, in a disturbance of the actions rather of the thoracic muscles, than of those which serve for articulating the vocal sounds; the latter lesion being only secondary and consecutive.

By the hurried and precipitate expulsion of the air in too large quantity from the chest, the sonorous waves, which serve for the formation of the voice, and which are about to be modified by the peculiar configuration that the buccal cavity must assume for each letter, are deranged and become confused. The muscles of articulation do not contract with readiness and ease, but are 'genés' in their movements; hence the great difficulty of pronouncing certain syllables, the more or less frequent repetition of certain others, and so forth.

From this theory of the production of Stammering springs a very obvious indication for the treatment of it; viz. to prevent the expulsion of the air 'en pure perte' from the chest during the act of speaking. The chief difficulty experienced by the stammerer consists in detaining the air in the chest, and in allowing it to escape only very slowly and gradually. To effect this object, it is especially necessary to attend to the following rules in speaking: first of all, to make a gentle inspiration, as in the healthy state; then to make a very slight pause; then to begin to talk, taking especial care to keep *the chest continually somewhat dilated, and the abdomen slightly protruded,* giving out, all the while, as little air from the chest as possible; and lastly, before recommencing the same series of movements, to expel the air that remains behind by a *powerful expiration.* The most important point for the stammerer to attend to is to keep his abdomen slightly saillant—a position which, by causing the descension of the diaphragm, forces the chest to remain expanded. M. *Becquerel* always observed the necessity of attending to this injunction in his own case.

M. *Jourdant* generally advises his patients to mark with their thumb the three several acts which he wishes them to attend to—viz: 1, the inspiration and the rest; 2, the articulation, while the diaphragm has descended, and the chest is dilated; and 3, the expulsion of the air, that remains behind in the lungs, by a strong and forcible expiration. The greater the quantity of the air that remains in the chest for the final expiration, the better. If these simple rules be strictly attended to, it will be found that no stammering can take place, even if the person tries to do so. It requires indeed assiduous efforts on the part of the stammerer, and these too continued for several days, before he can hope to overcome the difficulties in question. Once however that he succeeds in fully understanding and reducing to practice the mechanism of the treatment, he can pursue the plan now recommended at his leisure with increasing facility and effect; for the respiration gradually becomes less and less embarrassed, and the muscles, both of the chest and the vocal organs, become more obedient to the dictates of the will. As a matter of course, we cannot anticipate much benefit from this, or indeed from any other, method of cure, till the individual is old enough to have due command over his feelings and resolutions.—*Traité de Begayement, &c. par Dr. Becquerel.*

---

### On the Cause of the Lassitude and Panting in Ascending High Mountains.

It has invariably been found by all travellers, who have ascended very lofty mountains, that, at a certain elevation, any muscular exertion brings on very speedily a suffocative panting of the breath, and so complete a prostration of the muscular strength as baffles the stoutest heart to overcome. For example, we learn that *Saussure,* on the top of Mont Blanc, could not advance more than 12 or 15 paces at a time, before he was obliged to stand still and even to sit down, in order to take his breath and recover his strength. When he was at rest, the only inconvenience that he experienced was a sense of slight oppression in the region of the heart—which the moment before had been beating with great rapidity, but was now tolerably quiet. On the slightest movement however he was forced again to stand still, and pant for some minutes, with his face turned to the wind. The guides, who attended the philosopher, were similarly affected.

Now, the question comes to be, how are we to account for this extreme embarrassment of the breathing and this utter powerlessness upon exertion? Hitherto no very satisfactory explanation has been given of these physiological facts. M. *Brachet* of Lyons has recently suggested the following hypothesis. "It is not," says he, "the mere rarefaction of the air that occasions the lassitude and anhelation in the circumstances mentioned, since these phenomena are, without changing the air, either not produced at all, or quickly cease to exist, when the person is at rest. Neither can it be any real fatigue that occasions them; as they are experienced the very moment that the traveller re-attempts to walk, after he has rested for a length of time, and when he seems to have quite recovered his expended strength, so that he perhaps fancies himself able to make a considerable advance.

"Assuming the principle that the arterial blood becomes of a darker and darker colour, while traversing the muscles, in proportion as these are in a state of more powerful contraction, we are readily led to the following satisfactory explanation. During the state of rest, the venous blood returns to the heart less disoxygenated than usual, and consequently it requires a less quantity of oxygen, in its passage through the lungs, to give it a vermilion colour, and the necessary amount of stimulus. On any muscular exertion however taking place, the returning blood, being then of a darker colour, has need of a larger quantity of oxygen than before. Now as the air, being in a state of very great rarefaction, cannot afford a large supply of the vital gas at once, it is necessary to make up for this deficiency by an increased frequency in the acts of respiration: hence the panting and dyspnœa that are felt. As to the lassitude that is experienced, it is only, so to speak, the phenomenon inverted, the effect become the cause. It is well known that, if the arterial blood is rendered dark, the muscles, to which it is sent, will then not receive the full amount of stimulation required, and their power of contraction will be proportionately feeble. This is exactly what takes place in persons on the top of a high mountain; the blood, made black by its passage through the contracted muscles, no longer meets with a sufficient quantity of air, in the vesicles of the lungs, to restore to it its necessary vivifying stimulus. It therefore returns to the muscles, as well as to all the other organs of the body, less and less highly oxygenated: the results of which condition are the atony of the muscular energies, and the extreme lassitude of the body."—*Journal de Medecine de Lyon.*

*Remarks.*—The interpretation given by M. *Brachet* is ingenious; but let it be observed that it does nothing more than simply afford a very plausible explanation of the mode, in which the extreme rarefaction of the air at high altitudes—the essential cause of all the distress experienced—may be supposed to act on the state of the breathing and of the circulation. The observations of Aeronauts have abundantly shewn that, when the body is in a state of perfect quietude, the respiratory process is but very little disturbed. It is only when any muscular effort is made—for then there is obviously a greater consumption of oxygen—that fatigue and breathlessness are experienced. We see an illustration of the same fact, wherever the breathing is from any cause embarrassed or impeded, as in Asthma, threatened Suffocation, &c. The greater the struggles that are made with the limbs, the greater is the distress and the danger: safety under such circumstances consists in the quiescence of the body, and in the moderated action of the lungs.

---

### Hygienic Effects of Swimming.

There is very little doubt but this most healthful and invigorating exercise is far too much overlooked, in the present day, as a most useful and potent means of hygienic and therapeutic treatment. In not a few chronic maladies, a cure is to be expected, not so much from the administration of any drugs or medicines, as from a regulated regime in reference to diet and exercise. It is always better to evoke, so to speak, the restorative and healing energies of Nature—that may have become, as it were, dormant from want of being called into play—than to trust to the foreign and extraneous operation of man's interference, in our attempts to restore the body when feeble, and animate the mind when sickly or oppressed. Now, in no manner can this be so effectually done as by the steady and regular employment of gymnastic exercises; and of these, perhaps, none is on the whole equal to that of Swimming. All hardy and simply-living nations have borne testimony to the truth of this observation.

The Greeks and Romans fully appreciated the advantages of regular swimming exercise. In the time of Augustus, a man, that could not swim, was held in contempt. This Emperor ordered that as much attention should be paid to instruct his nephew in swimming, as in teaching him to read. The Spartans were obliged, by their military laws, to exercise themselves every day in the Eurotas, and the Roman soldiers were enjoined to do the same in the Tiber. These nations attached a very prominent importance to swimming, among the gymnastic exercises of the state, being well aware how admirably calculated it is to give strength and tone to the limbs, and to impart pliancy and facility of movement to the body. In this they were right; for in truth the act of natation calls into energetic play almost every muscle of the frame, without being liable to induce the troublesome consequences that not unfrequently accompany the exercises of running, leaping, riding, dancing, &c. All these latter exercises are apt to induce a more or less copious amount of perspiration—thus a considerable loss of animal matter takes place,—and may expose the person to a retropulsion of the fluids, and consequently to the risk of serious accidents. In swimming, however, there is no such liability; and, if the natatory movements are moderately and regularly performed, the play of muscular action is effected without distress; whereas, in other exercises, the movements are almost always apt to be excessive in certain muscles, while the rest are in a state of nearly perfect repose.

Medical men will do well to impress very forcibly on the attention of parents the excellent advantages of swimming to children, after the sixth or seventh year of their age. Independently of the moral benefit young people derive from the exercise, by diminishing their natural timidity, their physical health is thereby much strengthened and improved, and their body and limbs become more agile and graceful in all their movements. Fortunately, the Italian people have of late years began to appreciate the truth of this remark; for now we see the children of both sexes accustomed, in early life, to practise the healthful exercise of swimming. It is especially useful to such children as are of a lax fibre and scrofulous constitution; often have we had occasion to observe the gradual dispersion of abdominal fulness in such habits, under the steady use of swimming exercise. Sea-bathing is unquestionably to be preferred, more especially in such cases; but, if this cannot be obtained, let not the exercise be neglected, when a fresh-water bath can be procured.—*Guastalla, Studii Medici sull' Acqua di Mare,* 1843.

*Remarks.*—It is almost quite unnecessary to add a single word in commendation of Swimming, especially in salt water, as an important part of hygienic regime. Every young person ought to be taught to swim; and well would it be for many delicate girls, if false and foolish notions of decorum, on the part of their parents, did not too often debar them from using this most invigorating exercise. It should be more especially recommended, when there is any tendency to spinal weakness or lateral incurvation. The regular and equipoised exertion of the muscles on both sides of the back cannot but be beneficial in rectifying the incipient infirmity.

---

### On the Post-natal Descent of the Testicles, and the Accidents thereby occasioned.

The subject of the following remarks is but little known or understood by the majority of medical men. *Hevin* and *Hunter* are the only writers, who have endeavoured to determine the age at which the testicles usually descend into the scrotum, in cases where they were still within the abdominal ring at the time of birth: the former asserting that this evolution generally takes place about the period of adolescence, and the latter, at some time between the 2nd and 6th years of life. Though there may be some difference of opinion on this head, the main fact, however, that the testicles do descend at some time or another subsequent to birth, is admitted without hesitation by both these observers. Now this is the very position that M. *Oustalet* has undertaken to disprove. Resting his opinion on the results of his own experience—which too has been enriched not a little by analogous observations collected by his father—this gentleman maintains that, in a very large majority of cases, the testicles, when arrested within the abdomen at the time of birth, do not descend at all, at any subsequent period, into the scrotum.

In support of this assertion, the author relates 16 cases, as examples of the accidents—occasionally grave and severe—which are apt to be induced by this state of things: in almost every one of these cases, the patients had passed the period of adolescence. The symptoms and effects observed were stiffness and embarrassment in the movements of the limbs and body, engorgement and sometimes a veritable strangulation of the testicles, scirrhous degeneration of the seminal glands, and tendency to the descent of the bowels along the inguinal canal.

After comparing the inconveniences and dangers of these accidents on the one hand, and of the persistence of the testicles within the abdomen on the other, M. *Oustalet* comes to the practical conclusion that, "when we discover at birth that one or both testicles are still within the ring, a bandage should be at once applied, and every means employed to induce a speedy obliteration of the abdominal aperture."—*Gazette Medicale de Strasbourg.*

*Remarks.*—It is objected, by a French reviewer of this memoir, that M. *Oustalet* has not very satisfactorily established the propriety of the practice he recommends, because he has failed to shew that the number of patients, in whom the arrest of the testicles does really induce troublesome consequences, much exceeds that of those in whom this anomaly causes little or no inconvenience. Let us see how the truth stands in reference to this matter. *Wrisberg* has calculated that, of every 102 fœtuses at the full term, there are not fewer than 12 in whom the testicles have not yet come down. Now, if every medical man will consult his recollections of practice, it will readily be admitted that the number of cases, in which the accidents described by M. *Oustalet* have been observed, is very far short of the ratio of 12 to 102: and yet this would necessarily be the case, to justify the apprehensions and consequently the practice of this gentleman.

---

### ON THE UTILITY OF SURGICAL OPERATIONS IN CANCEROUS DISEASES.

The grand points of this most important surgical question are to determine—1, if it be really true that Cancerous disease is primarily of a local nature, and subsequently degenerates into a constitutional malady; and 2, if extirpation, performed at an early period, prevents the occurrence of this degeneration. With the view of elucidating these matters, M. *Leroy d'Etiolles* has collected the following statistical observations. Of 801 operations, 117 were performed within a twelvemonth after the first manifestation of the disease.

Of these 117 cases, there were 61 in which the disease had returned at the time when the reports reached me. It is more than probable that this proportion would be found to be still higher, if we knew the actually present state of these cases.

The results of operations for Cancer of the Lip are curious and worthy of notice, in consequence of the difference in this respect observed in the two sexes. Of 633 cases of Cancer in the male subject, 165 were examples of Cancer of the Lip: of these 114 were treated with the knife—12 with caustics. There were

15 relapses in all; that is, about an eighth of the whole. On the other hand, of 2,148 cases of Cancer in the female, there were only 34 instances of the disease in the lip: of these, 22 were treated by excision; and in seven—nearly a third—there was a return of the disease.

This difference does not hold good of Cancer of the Tongue; for then the disease is equally fatal in both sexes. Of nine operations, in which a cancerous tumor of this organ was extirpated, three were performed within one twelvemonth after the earliest appearance of the disease. In the other six cases, the patients died, the disease having previously returned.

As respects Cancerous diseases of the Mamma, we find the following data. Of 277 operations, 73 were performed within the last two years: as yet we cannot say positively what are the results. Of the remaining 204 cases, 22 of them proved fatal in the year after the operation, and in 87 others there was a relapse of the disease.

M. *Leroy* deduces the following conclusions from his researches :—

1. Extirpation does not arrest the progress of Cancerous disease.
2. This operation should not be resorted to, as a general method of treatment, except for Cancer of the skin and lips.
3. There is no necessity to extirpate Cancerous disease of other organs, except when an alarming hæmorrhage supervenes.—*Comptes rendus.*

The Academy appointed MM. *Roux, Velpeau,* and *Serres,* to report upon this communication of M. *Leroy.*

---

### M. Orfila's Reply to his Calumniators.

In the course of the present month (May) M. *Orfila* has published, in the French journals, a letter addressed to his *Confreres,* in defence of his conduct against a long-continued series of calumnies, with which he has been assailed for the last ten years (he says), not only as Dean of the Faculty of Medicine, member of the royal council of Instruction, and author of various works, but also as a dishonest and pretended political character. As this exculpatory epistle is viewed, by many of his countrymen, "as a document useful and important for the scientific history of the present age," it may deserve some notice at our hands.

He is accused, in the first instance, of base subserviency to government on numerous occasions, and more especially on that of his mission to *Blaye,* in 1833, for the unworthy purpose, his accusers say, "of officially ascertaining the dishonour of a captive woman—the Duchess of Berri, who was, at that time, a prisoner in the Castle—and even of using force in this unmanly office." We need scarcely say, that the charge in question is utterly without foundation, and must have arisen only from motives of personal hostility, or of base mendacious envy. The fact is, that M. *Orfila,* in company with MM. *Andral, Fouquier,* and *Auvity,* was appointed by the then existing ministry, of which Marshal Soult was at the head, to proceed to Blaye, to report upon the state of health of the Duchess, as it was currently reported at the time that she was very seriously ill.

On their arriving at the fortress, they found indeed that she was suffering with a pulmonary catarrh; but the symptoms were not at all alarming; and the medical men, therefore, did not deem it necessary to remain longer than was necessary in order to draw up a report of her case, and consult with the Duchess's ordinary medical attendant, on the treatment to be pursued.

M. *Orfila,* it seems, was sent on a subsequent occasion, to visit the illustrious *detenue,* about a fortnight after her accouchement, in consequence of its having been again publicly alleged, that her health had suffered very much from the imprisonment, to which she had been subjected. She refused, indeed, this time to see the medical men—that had been sent by Government from Paris; but the sole motive for this refusal was, it seemed, because she was quite satisfied with her own private physicians, and she moreover now declared that she would receive no visits from any strangers, unless she was also permitted to see her personal friends, MM. *Chateaubriand* and *Hennequin*. She, however, wrote a letter to M. *Orfila*, full of kind expressions, and conveying her regrets that circumstances prevented her from admitting him to her presence. Such are the actual circumstances of a mission that has been rashly and unjustly pronounced by his accusers, to be " dishonourable and utterly unprofessional."

It is unnecessary to notice the charges that have been brought against M. *Orfila*, in his capacity as Dean of the Faculty of Medicine and Member of the Council of Education, as we have no means of exactly ascertaining the truth or fairness of them; nor, even if they were substantiated, can the subject at all interest the foreign reader. We observe, by some of the French Journals, that one of his capital offences is, in having been accessory to the appointment of M. *Guerin* as surgeon to a public hospital. This was probably an injudicious step; but surely it can never justify the unmeasured condemnation which is sought to be heaped upon him. However this may be, we pass on to his defence against the very serious charge of prostituting his scientific talents in the alleged following manner.

*One of the most grave reproaches with which history will have to charge M.* Orfila, *is that of having perverted the duty and character of a medical man when appealed to by justice, of having accustomed the tribunals to regard him always as a sort of necessary adjuvant to the accusation, of having at pleasure transformed his mission of pity and charity into something which, in hands not so well intentioned as his own, would hold the mean between an accuser and an executioner. However, to be just in all things, we willingly state that, on one occasion, M.* Orfila *had to change his accustomed part; and that he is fairly entitled to the high distinction of having saved an innocent person from the scaffold: we allude to the well-known affair of* Chambery.

" I was not aware," replies M. *Orfila*, " that the mission of a legal physician was a mission of pity and charity; I had supposed, with all honourable men, that the *savant*, when consulted by the tribunals of justice, should restrict himself to the simple enunciation of the truth.

" Thus, because my investigations have led to the detection of crime on ten different occasions in the course of my medico-legal career, and because my evidence on these occasions was fatal to the accused, I am stigmatised as something between an accuser and an executioner! Since, too, the writer of the article against me has determined on not bestowing any praise, except for those cases in which science is instrumental in saving the accused from condemnation, I may fairly be permitted to claim some for my conduct on the following occasions, which he has, no doubt unintentionally, omitted to mention.

" In 1826, the body of a young man of the name of *Alberici*, a Carbonaro of Lombardy, was taken out of the Po. The physician, commissioned by the government to report on the case, gave it as his opinion that the man must have been thrown into the water, *after* he was strangled. Five of the national guards, who had been sent to lead *Alberici* to prison, were accused of the murder, tried, and condemned to death. Confident of their complete innocence of the charge, these men petitioned for a new trial, and submitted their case to various learned societies in Italy.

" In consequence of the discrepancy in the judgments that were received, I was requested by the counsel for the defence to investigate all the particulars, and to state my professional opinion of the case. After examining all the documents and evidence connected with the accusation, I drew up a memorial, which concluded in these words: ' that it was certainly rash to affirm that *Alberici* had been strangled, that nothing in the documents of the cause warranted this opinion, and that the report of the post-mortem inspection had been drawn up with an inconceivable remissness.'

"My efforts were crowned with entire success; for the already convicted criminals were straightway acquitted.

"2. A woman, of the name of *Trichereau*, accused of having poisoned her husband, had been kept in prison for nine months, on suspicion of having been guilty of that crime, when I was desired, in company with M. *Lesueur*, to examine and report upon the case. As soon as our report was laid before the attorney-general (procureur du roi) the accusation was abandoned, and the woman set at liberty.

"3. *Louise Lannier*, accused of infanticide, was tried before the Court of Assizes of the Seine, in July 1842. Her mother, also, was suspected of having been privy to the crime. Two medical men, that had been charged with examining the affair, gave it as their opinion 'that the death had been caused by the immersion of the infant in the fluid of a privy,'—in consequence of their having found a similar coloured fluid in the minute ramifications of the bronchi. I was consulted by the counsel for the accused, as to the value of the medical evidence that had been given, and the following is a summary of my answer:—'The question, whether fluids, in which a dead body has been found immersed, may penetrate, *after death*, into the minute bronchial tubes, has long been the subject of lively discussion. To ascertain the truth, I have made numerous and very varied experiments, sometimes on human corpses, and at other times on dogs, and other animals; and the result of all these researches is, that I have come to this incontestable and uncontested conclusion, that the *presence of fluids in the extreme bronchi does not constitute a proof of submersion during life*,—since, fluids have been found to penetrate quite as far into their ramifications when the body had been immersed immediately, and even several hours, after death had taken place?' The accused were accordingly acquitted.

"4. I do not exaggerate, when I say, that there exist, at this present moment, in the Archives of the Royal Court of the Department of the Seine, more than thirty reports, almost all written in my own hand, on different cases in which my opinion has invariably and at once effected the acquittal and liberation of the prisoners.

"This part of my defence I cannot more appropriately close than by quoting the profession of faith, which I made in 1840 before the Court of Assize at Dordogne: 'I have detailed at considerable length the scientific facts of the cause in question, I have furnished all the information that has been required of me, and I am still ready to communicate whatever I know or may be desired; but let it be remembered that I am not here to speak in support of the accusation any more than in the interest of the accused; *my mission is one solely of science and of truth.* In my opinion, the last words pronounced in this Court ought assuredly to be in favour of the accused. A medical man would utterly mistake his duty, if he endeavoured to weaken or invalidate the defence, when the accused, already borne down under the weight of crushing charges, can retain no hope of escape unless this (the defence) retains all its force and full impressiveness. It belongs to the public minister to reply, and avail himself, if he thinks proper, of all the facts of science that may serve his cause: to every one belongs his own peculiar office and duty.'

"Could language like this be expected from a man who is designated as 'something between an accuser and an executioner'?

"The last charge on this head, that has been made against me, is couched in these words: *On the occasion of every new experiment, we have seen M.* Orfila *deciding the greatest difficulties of science with the same air of assurance, the same bold tone of affirmation, although perhaps, only a few years before, he was in the habit of employing means of investigation altogether different from what he em-*

*ploys at the present time. We are indeed distressed to see one of the highest personages of one profession compromise the value of science and the dignity of our art by rash unguarded assertions and a presumptuous assurance, which might at once vanish on the discovery of a less fallible re-agent than has been hitherto used.*

"The whole of this statement is utterly false. In my investigation of the different mineral poisons,—for it is obviously to this subject that the allusion in the foregoing passage is made,—from the time that I first pointed out the necessity of destroying all the organic matter with which they might be blended, and the mode of effecting this, I have *constantly* made use of either the nitrate of potass, or the nitric acid; and moreover, I have always had recourse to sulphuretted hydrogen (l'acide sulfhydrique) or to Marsh's apparatus, for the purpose of discovering and making apparent the presence of the poisonous matter. Without doubt, this latter instrument has been improved since it was first introduced; but does this circumstance at all warrant the inference that the Arsenic, which was thereby discoverable when it was less perfect, is now not arsenic at all? Do we not rather see that, so far from injuring the cause of the accused, the improved apparatus rather tends to favour it? It is especially unjust to me not to acknowledge that, since the publication of my work, I have been the first to suggest and discuss, in special memoirs, the various objections which might be made to the system which I have created, and that I have thus myself furnished weapons to serve the cause of the accused. These objections relate especially to the impurity of the tests employed, to the nature of the different soils which may be impregnated with Arsenic, to the imbibition of poisonous matter, &c. Ask the advocates of the accused if they have ever combated my reports by any other means, save those with which I have supplied them in my own writings." —*Gazette Medicale.*

---

### M. MITSCHERLICH ON FERMENTATION.

The act of Fermentation is produced by the formation of a Vegetable being, just in the same manner as that of Putrefaction is by the formation of an Animal one. The infusory animalculæ, on which the latter process depends, have been described as consisting of one or of several minute globules, grouped together side by side, and endowed with a sort of creeping or rotatory motion. According to our author's observations, the other animalcules, present in putrifying substances, are to be regarded as having been deposited in them from the air, either by insects, or in some other way which we do not so well understand. * * * * A certain quantity of Oxygen is necessary for the due development and conservation of the Vibrios, that are found to exist in animal bodies. These animalcules are observed to be abundant throughout the entire extent of the alimentary canal, from the mouth to the anus. One of the readiest means of detecting their existence is to examine with the microscope the inter-gingival deposit, or matter which accumulates between the teeth. Occasionally they may be perceived on the surface of the skin; but M. *Mitscherlich* has never detected them either in the blood or in any of the secretions. * *

If a small portion of sugar be added to a fluid which contains these animalcules, not only do they continue to form in great numbers, but there is produced at the same time a vegetable product—viz: the ferment or Yeast. If more sugar be added, the formation of the animalcules is impeded or even entirely suspended, while the generation of the former becomes more abundant.

In a clear fluid, in which fermentation is about to commence, we observe first of all that it becomes somewhat troubled; and we discover, with the aid of the microscope, the presence of numerous globules of different sizes. Day by day these globules become larger and larger, while new ones are continually forming at the same time. In some fermentable fluids, as in grape-juice, these globules usually remain single and isolated, the one from the other; in others, the globules enlarge in bulk and give off new ones from their sides, in the same manner as buds sprout from various vegetable forms. There is indeed a marked difference in this respect in the upper and lower yeasts, that exist in the fermented liquor of breweries: in the former, the globules are generally composite and aggregated; while, in the latter, they are almost all solitary and detached. The globules consist of a vesicular envelope and of granular matter within; the separate granules being the *Spores,* or germs of future globules. * * *

Substances, which exercise a poisonous action on the Fungi, are also found to destroy the working effects of Yeast: corrosive Sublimate may be adduced as an example. On the contrary, those substances which act energetically on the animal organism—such as the Emetic (Ipecacuan ?) in the solution of which the fungi are so quickly formed—have no effect on the process of fermentation.

A great number of the Fungi, which are known as diseases or morbid growths on vegetables, have a similar agency with genuine Yeast: they give rise to great and important changes in the composition of the surrounding organic matter. Thus it is with the Fungi of trees in reference to the ligneous fibre.

These and other analogous facts unquestionably open up a new field of enquiry as to the various compositions and decompositions, which the roots of plants may produce in the soil.

The process of Fermentation presents therefore a multiple and very varied interest. It is by the development of a substance of contact, so to speak, that the decomposition of one of the most important chemical combinations is effected. This substance is now found to be an organised living existence; it belongs to one of the simplest forms of animate development, the successive phases of whose evolution may be most satisfactorily traced.—*Journal de Pharmacie,* Sept. 1843.

---

### Process of Embalming.

The method pursued by Drs. *Broc* and *Pougin* in embalming the body of Marshal Count d'Erlon—one of Napoleon's most distinguished officers—who recently died in Paris, was the following. A mixture, composed of a Solution of Corrosive Sublimate in alcohol (500 grammes of the salt in 2000 grammes of fluid), of a Solution of Arsenic in water (25 grammes in 250), and of a Solution of Aromatic Essential Oils in spirit (24 grammes in 2000), was injected into the lower end of the left common Carotid Artery. A portion of the mixture was also introduced into the Cavities of the pleura and peritoneum, the gaseous contents of the intestines having been previously liberated by numerous punctures in their coats. The body was afterwards enveloped in a multitude of linen bandages. As a general remark, it may be stated that not more than about three *litres* of fluid altogether should be injected into the body of an adult; otherwise it is apt to exsude into the bronchi, and to return by the mouth in great abundance.

---

### The Neapolitan Phlebotomist.

The following description of the *Salassatore* of Naples is from the pen of a recent traveller in Italy, who has published what he calls his "Medical Impressions," in the *feuilleton* article of the Gazette Medicale.

"How often have I stood before the marvellous sign-boards, over the blue-framed windows of the shops of these ministers of the lancet! Imagine to yourself a man, painted in all the naiveté of the nudities of a terrestrial paradise, and discharging from every vein, which the lancet can possibly reach, parabolic jets of blood, whose streams inundate the floor. Then picture to yourself besides the phlebotomist himself, with instrument in hand, and kneeling in admiration of his work, like Pygmalion before the statue which he had made, and you will have a complete idea of the manner, in which the *Salassatore* announces by the aid of pictorial art the various results of his skill. I was curious to come in contact with one of these professors of Minor Surgery, and accordingly went into his shop on the pretext of asking for leeches; for the *Salassatore*, as is most right, deals in these adjuvants of his art. He was at the further end, gravely stretched out upon a straw couch, awaiting the entrance of a customer, with that sleepy, do-nothing calm—the 'dolce far niente' sort of air—which is so characteristic of the Neapolitans. While he was engaged in taking out the leeches from the glazed earthen vessel in which they were kept, I cast my eyes round to observe the contents of the shop. It was very poorly furnished on the whole; but there was a profusion of little cases full of bandages and compresses, that were ranged along the walls from the top to the bottom. I ventured to make an enquiry about them; he told me that each compartment represented a subscriber, who came at regular and stated times to be bled; I drew back in astonishment at the bloody statistic intelligence that was given me; and, for the first time in my life, rendered justice to the *moderation* of our French advocates for bleeding 'coup sur coup,' only in cases of sickness."

---

### ON THE TREATMENT OF BUBOES.

As long as inflammatory symptoms are present, the usual treatment by leeches, poultices and so forth, is to be steadily pursued. When suppuration has been once established, I generally apply to the centre of the bubo a small blister, (of the size of a sixpence or shilling), the vesicated surface being afterwards kept moist with a pledget of lint dipped in a solution of Corrosive Sublimate—1 part of the salt in 32 parts of distilled water: this is kept on for two hours or so, until a superficial eschar has been formed. When this has been done, an emollient poultice is to be applied to promote the decadence of the eschar. Care should be taken that the eschar is not too deep, and that it has not destroyed the entire thickness of the dermis. The inclosed purulent matter oozes out through the pores of the latter, when the eschar falls off, and the dermoid tissue becomes agglutinated (la peau se recolle). When the bubo has opened and begins to discharge, and sloughs present themselves, I usually dress the wound with lint that has been dipped in honeyed wine; and if this plan does not answer well, I inject under the detached skin a weak solution of the Sublimate. In the event of this treatment failing, I have recourse to small blisters, which very generally have the desired effect of causing the agglutination of the sides of the wound. It is very rare that I have found it necessary to excise the loose integuments. When the edges of the abscess are effaced, and nothing but an ulcerated surface exists, the healing process will often be found to be promoted by the use of the Styrax ointment, the balsam of Arceus, the occasional application of the lunar Caustic, or of the acid nitrate of Mercury, &c. M. *Regnaud* speaks favourably of the Creosote ointment—which in his opinion is decidedly preferable to the powdered cantharides, as recommended by M. *Ricord*—in many cases.

When the bubo is in a state of chronic induration, he has used, he tells us, with most advantage, the pommade of the proto-ioduret of Mercury (3—5 parts to 30 of lard): blisters rank next in his estimation. He has occasionally employed, with good effects, the application of heated bricks, as recommended by M. *Henrotay* of Antwerp. The bricks, after being duly heated in a stove or oven, are enveloped in flannel and kept on the swelling by a bandage, until they cool, when they are again heated and re-applied.—*Bulletin de Therapeutique.*

*Remarks.*—The practice here recommended, by a surgeon too who must have had a very large experience from the post he has held for a length of time in the Marine Hospital at Toulon, seems to us to be very needlessly complicated. When the suppurative stage has commenced, might not the application of the caustic potass, so as to form an eschar of appropriate depth, supersede the more tedious operation of first blistering the skin and then applying a corrosive stimulant to it? Unquestionably the best method, as a general rule at least, of opening bubos is with caustic,—in preference to the use of the knife—and we are therefore glad to find that M. *Regnaud* is in the habit of adopting it: the only objection we make to his practice is as to the mode of effecting this purpose. When the abscess is once fairly open, and its surface and edges are in an unhealthy condition, few applications are so good as the Tinct. Benzoini Comp. (Friars Balsam) applied with lint: it is an admirable detergent to foul ulcers in general. Besides its stimulating and antiseptic properties, the balsam seems to act beneficially by totally excluding the admission of the air to the sore, in consequence of the varnished coating which it forms. When the ulcer is tolerably clean, but very tardy in healing, we have seen excellent effects from covering it daily with a layer of powdered rhubarb. As a matter of course, bark and other tonics are generally required to be administered internally at the same time.—(*Rev.*)

---

### On the Treatment of Secondary and Tertiary Syphilitic Diseases.

M. *Devergie*—one of the physicians of the St. Louis Hospital, which receives a much larger number of syphilitic patients than any of the other hospitals in Paris—has, for the last eight years, met with great success, he informs us, in the treatment of the secondary and tertiary affections by adopting the following remedies. The patient is to drink every day about a quart of a sudorific ptisan, in which from five to twenty grains of the Ioduret of Potash have been dissolved, and also to take every morning, fasting, a pill composed of guiac, opium and a minute quantity of the corrosive sublimate. In the course of a week or so, a second pill is to be taken at night also. These medicines are to be persevered with for two or even three months, without intermission. A tepid bath is to be taken once a week. No wine is allowed; but milk is given freely instead. If the patient's constitution has been much damaged by irregularity or want, M. *Devergie* recommends the use of some ferruginous preparation, or of bark, or of both together; at the same time diminishing the dose of the sublimate, if the state of the symptoms should still require its continuance.

When, after six or seven week's use of these remedies, the local symptoms still exhibit an unhealthy character, the application of the crystallized acid nitrate of mercury—dissolved in water, to which a few drops of nitric acid have been added—will be found most convenient. The ointment of the proto-ioduret of mercury is also a valuable application, to promote the healing of certain ulcers.

---

### Applications of Subcutaneous Surgery.

The most remarkable feature of subcutaneous surgery is the speedy and genial cicatrization of divided tissues, without any accompanying inflammatory re-action, or tendency to suppuration. Another important character, that deserves to be attentively considered, is the readiness with which many fluids, when effused into the cellular texture, are absorbed and entirely removed, if the access of the atmospheric air be prevented.

The applications of Subcutaneous Surgery may be reduced to two heads; viz: to sections, and punctures; in other words, to operations on the tissues, and to those on sacs and collections of fluid. From this two-fold view arises the consideration of the *non-inflammation* of the divided tissues on the one hand, and of the *unalterability* of effused fluids on the other. M. *Guerin* enumerates in the following catalogue the numerous applications that have been proposed, either by himself or others, to the various maladies and injuries of the different structures of the body.

1. The Skin.—The 'decollement' of the skin in cases of vicious scars and adhesions.

2. Tendons.—The section of these—*a*, for deformities; and *b*, for the purpose of facilitating the reduction of old dislocations, and of recent fractures.

3. Aponeuroses.—The section of these—*a*, as an orthopædic means of cure; and *b*, as a means of relieving the tension of the tissues in various inflammatory engorgements and effusions.

4. Muscles.—The section of these—*a*, as an orthopædic means; *b*, as a means to favour the reduction of luxations and recent fractures; *c*, for the purpose of facilitating the operation for strangulated hernia; *d*, for the radical cure of irreducible hernia; *e*, the section of the sphincter of the anus in cases of fissure; *f*, the subcutaneous cauterization of the muscles as a means of artificial contraction.

5. Ligaments.—The section of these—*a*, as an orthopædic means; *b*, as a means to facilitate the reduction of certain luxations and fractures; *c*, the cauterization or calefaction (?) of the ligaments, with the view of strengthening and contracting them.

6. Arteries.—*a*, The subcutaneous scarification or cauterisation of certain fungous tumors; *b*, the obliteration of arteries by section, puncture, or scarification; *c*, their subcutaneous ligature.

7. Veins.—*a*, Their subcutaneous section and scarification in the various kinds of varicose swellings; *b*, the ligature of the veins.

8. Lymphatic Vessels and Glands.—*a*, Section of the former to promote abortive treatment in buboes; *b*, section and scarification of the latter, affected with chronic tumefaction.

9. Nerves.—*a*, The section of these in cases of neuralgia; *b*, the scarification of their minute branches in cases of sharp pain under the skin; *c*, the cauterisation or calefaction of them in certain painful affections, as in arthralgia, &c.

10. Cartilages.—Subcutaneous symphyseotomy.

11. Bones.—*a*, The removal of small exostoses; (!) *b*, subcutaneous fractures to remedy certain deformities caused by irregular callus; (!) *c*, the extraction of sequestræ, and the resection of the sharp ends of fragments of a fractured bone, that threaten to force their way through the skin; *d*, scarification of the surface of a bone when tumefied and inflamed; *e*, cauterisation or calefaction in similar circumstances; *f*, extirpation of osseous tumors.

Besides the above long catalogue of the applications of Subcutaneous Surgery, M. *Guerin* enumerates a variety of others, belonging to the second class of its applications, as the puncture and incision of incipient phlegmonous swellings; the puncture and evacuation of meliceric cysts, of chronic abscesses, of sanguineous tumors, serous deposits, of articular dropsy, &c.; the extraction of foreign bodies from the joints; the radical cure of Hydrocele and Hematocele; the puncture of the head in chronic Hydrocephalus, and of the dorsal swelling in Spina bifida; the puncture of the thorax in Empyema, and of the pericardium in dropsy of this sac; the puncture and evacuation of tumors of the liver, ovaries, and other abdominal cysts; and lastly, "a multitude of other operations, which cannot be performed either by section or puncture, properly speaking; but which nevertheless may be reduced, in a certain degree, to the principal condition of the method in question, and which may be benefited by its peculiar advantages. Among these applications, I may mention the Cæsarian Section." —*Gazette Medicale.*

*Remarks.*—Like all modern discoverers and inventors—if indeed he is entitled to be ranked as such—M. *Guerin* greatly exaggerates the value and importance of his offspring. Modesty is certainly not a character of the present age; and no set of men seem to have so little of this grace as the doctors, when they are so fortunate as to hit upon anything that is somewhat novel, or to make a new application of an old and well-known remedy. Who has not heard of chronic inflammation applied to the history of almost every disease to which the human body is subject, until nosology was deemed an empty labour, and the science of therapeutics was reduced to the application of a mere mechanical formula, which any chemist's shop-boy might understand quite as well as a *Sydenham* or a *Cullen?*

The truly valuable discovery of *Laennec* has suffered much more at the hands of its friends than of its enemies; and its extravagant laudation some years ago is beginning to give way to its undeserved disparagement in the present day.

Of later years, the doctrines of the reflex function and of the excito-motory actions, as they have been called, has been pushed by its propounder to a most wearisome length, until the profession has become sick of the endless iteration of the same thing in different guises. There is far too frequent a repetition of 'I' and 'my' in all his writings on the subject. M. *Guerin* is guilty of the same bad taste as our more talented countryman. He writes not as the expositor of truth or the interpreter of nature, but rather as an interested advocate in support of a cause which he has undertaken to uphold.—*Rev.*

---

### Use of Colomba-root against Vomiting.

This well-known and long-established drug is an admirable remedy in many cases of *ataxic* and *nervous* Vomiting. A writer, in a recent number of the Annali Universali, mentions the case of a soldier, who was suddenly seized, after a fright, with violent pain in the abdomen, accompanied with vomiting and diarrhœa. The other symptoms gradually ceased; but the vomiting (which was evidently of a nervous character) still continued. The patient had a yellow cachectic look, a furred white tongue, and loss of appetite; slight epigastric pain usually preceded the coming on of the fits of vomiting. They generally returned six or seven times every day. Every thing tried failed in checking this obstinate irritability of the stomach, until—seven months after the commencement of the disease—Dr. *Manfredonia* suggested the use of Colomba: it was given in the form of powder, and in the dose of eight grains four times in the 24 hours, in a small cupful of goats' milk. A very sensible amendment in the state of the patient speedily took place, but the vomiting did not entirely cease for some time. The Colomba was subsequently given in the form of the decoction; and under its use the health was gradually, but completely, restored.

(Colomba is an excellent remedy in a great many cases of gastric ailment: a favourite formula of ours is—Infusi Colombæ ℥vj., Liq. Potassæ ʒiss., Tinct. Humuli ʒiij., Aquæ Pimentæ ℥iss. M. It is often very useful in phthisical patients.—*Rev.*)

---

### TOBACCO-SMOKE APPLIED TO GOUTY LIMBS.

In M. *Reveillé-Parise's* work on Gout and Rheumatism, we read that "the fumigations of Tobacco—recently proposed by the Abbé *Girod*, canon of Nozeroy, and which consist in exposing the pained part to the smoke of the dried leaves, thrown upon heated coals, for about a quarter of an hour at a time—have been often found to afford great relief: they may be repeated three or four times in the course of the day. To guard against the return of the malady, the Abbé advises the occasional use of a foot-bath made by boiling tobacco-leaves in water."

The remedy is cheap, and may certainly be sometimes useful. Dr. *Hinard* reports very favourably of its soothing effects upon himself.

(Have any of our French brethren ever tried the homely remedy of cabbage-leaves—previously softened by steeping them in hot water, and then slightly dried—as an application to gouty and rheumatic joints? Often have we occasion to witness its good effects. Another very convenient and useful application is a wash composed of water, vinegar, and any spirit—from gin to eau de Cologne: it should always be used lukewarm, and the part should be covered with oil-skin.—*Rev.*)

---

### FALLACY OF THE NUMERICAL METHOD IN MEDICINE.

The following very sensible remarks, in reprobation of the mistaken importance attached by *Louis, Forget, Bouillaud,* and such writers, to one of their favourite doctrines, are from the pages of the Revue Medicale—the organ of the Hippocratic School in France.

" The principles of the Healing Art should be based on the discrimination of diseases, and not on their confusion. Now it is this very nosological error that the numerical system has given rise to; and with it at first the confusion, and subsequently the negation, of all genuine therapeutic medicine. Look where we are, now-a-days, with respect to Typhoid Fever—a term of such elastic pliancy as to admit any thing we choose; it is a sort of medical *goddam,* (Query, Is this a skit at English manners?); almost the sum-total of medical language of the present age. We are indeed quite well aware that the numerical system is not alone chargeable with all this confusion; Chemistry and Pathological Anatomy must come in for their share of the blame. If we would avoid the errors of our predecessors, we must be careful not to *base* the principles of the Healing Art on either of these sciences any more than on the Numerical System, that has been so much vaunted of late years. They, and indeed almost every branch and department of science, may render important services to practical medicine; but to seek to make any one its only basis, is assuredly a great folly: it is to make a principal of a mere accessory."

---

### LONG TUBULAR MEMBRANE EXPELLED IN A CASE OF CROUP.

In a recent number of the Brussels Journal de Medicine is narrated a case of Croup, in a child three years and a half old, where a membranous tube nearly five inches in length was expelled during the act of vomiting: on the surface of the tube were several reddish lines which looked like minute venous ramifications. The symptoms—which up to the time of the expulsion had been very alarming—immediately subsided, and the young patient afterwards rapidly recovered.

There was every reason to believe that the tubular membrane was the result of exsudation on the surface of the Trachea, and had become dislodged by the violent expulsive efforts of vomiting.

---

### Creosote, a good Application to Burns.

Creosote is one of the most valuable of those remedies which the ancient writers designated as *incarnatives ; i. e.* promoting cicatrisation. M. *Mascharpa* has drawn the attention of his countrymen (Gazzetta Medica di Milano) to its excellent effects in this respect, as an application to many ulcers. He has used it also in several cases of Burns with the most satisfactory results: it soothes the pain of the injury at the time, and accelerates the subsequent progress of the cure. The best mode of using it is in the form of lotion,—made by adding 20 or 30 drops of it to two or three ounces of water, and applied with pledgets of linen to the injured surface.

(The London Pharmacopœia, in its last edition, contains an "Unguentum Creosoti," prepared with half a drachm of the oil to an ounce of lard : it is applicable for the same purpose as the solution of the oil in water.)

---

### Antiseptic Properties of Creosote.

A solution of Creosote in water—10 drops to one *litre*—is an excellent antiseptic preparation for preserving anatomical specimens. It has the great advantage of not changing the colour or physical properties of the tissues immersed in it; and it also checks any commencing putrefaction or decay. Moreover, it does not act on the steel instruments. Pathological specimens, that have lost much of their elasticity and general form, by having been preserved for many years in alcohol, speedily regain their original aspect and characters, when they have been kept for three or four days in the Creosote Solution : and even dried preparations when treated so, will often resume the appearances which they had before they became shrivelled and contracted. The globules of Blood, Purulent matter, and other secretions may be preserved in this fluid, intact, either as to form or colour, for a length of time. M. *Pigné*, the assistant Conservator of the Dupuytren Museum, ingeniously, but perhaps rather fancifully, suggests that portions of the blood itself may be thus preserved, and alludes to the possibility of making a collection of hæmatological specimens, with the view of comparing the various morbid conditions of the circulating fluid.

---

### Ammonia in Delirium Tremens.

In one of the recent German Journals, we find a paper by a Dr. *Scharn* on the subject of this troublesome and distressing malady. The learned doctor having experienced no little disappointment from the use of the remedies in general use, and conceiving that, as the disease is nothing else but intoxication arrived at the period of its *apogee,* it should be treated by the very same means which are known to be most serviceable against the latter, had recourse to the employment of Ammonia, in the form of the pyro-oleaginous solution, or of the Succinate of the alkali. By means of this very simple and innocuous remedy he has succeeded, he assures us, in curing a great number of very severe cases of the disease.

It deserves notice that M. *Brachet,* also of Lyons—a gentleman whose opinions are justly entitled to consideration—has recently recommended the same remedy, Ammonia, in the treatment of Delirium tremens.

(Ammonia by itself will rarely suffice to subdue the excitement and allay the Restlessness of this Neurosis: Opium must almost always be associated with it. We have often used, with good effect, the Ammoniated Tincture of Valerian and the Liquor Opii Sedativus—not forgetting the application, at the same time, of warmth to the feet, and of cooling spirituous lotions to the head. When symptoms of febrile or inflammatory irritation are present, it may be necessary to resort to leeching, and the administration of effervescing saline draughts with Antimonial Wine).

---

### A FLOCK OF SHEEP POISONED BY EATING THE RANUNCULUS REPENS.

The sheep had not been many hours in the field before the shepherd observed that several of them seemed suddenly to fall down, as if they had been struck by lightning: their eyes rolled about in their sockets; their breathing was hurried and laborious; and some of them kept turning round and round as if they were dizzy, and died with their heads inclined over to their left flanks. He fancied that the seizure was owing to a 'coup de sang,' and bled the animals accordingly; but the loss of blood seemed to do harm rather than good, for eleven animals died almost immediately afterwards. A veterinary surgeon, who was summoned, immediately detected the cause of the mischief in the great admixture of the Ranunculi with the grass. He therefore at once recommended that the bleedings should be discontinued, and a dose of sulphuric æther in milk be given to all the affected animals. Under this treatment the alarming symptoms quickly subsided; and, although for some days the sheep remained very feeble and tottering on their legs, they all recovered completely.

---

### ANIMAL ELECTRICITY.

Signor *Matteucci* wrote to the French Academy to state that, after much trouble, he had succeeded in making a galvanic pile of five living pigeons! Both thighs of each bird were flayed; the exterior of a muscle in one pigeon was brought into communication with the internal part of the muscle in another; and so on in succession with all. In this manner he obtained a Galvanometer—which, in the first experiment, indicated 15° of a current, which was in every case directed from the interior of the muscle to the surface. The current diminished rapidly; so that, at the third experiment, a few minutes afterwards, it was not more than 6°; but still always in the same direction. The presence of effused and coagulated blood on the muscular surfaces is one of the causes of this diminution; for, on removing it, the current was observed to increase by a few degrees. The greatest difficulty, that was experienced in conducting the experiments, was to maintain the parts in contact: Signor *Matteucci* used wooden forceps and ligatures for this purpose.

The muscular electrical current increases in intensity with the degree that the Animals, experimented upon, occupy in the Zoological Scale—a circumstance that seems to prove its chemical origin; or, more correctly speaking, its connexion with the chemical actions of nutrition and the transformation of the tissues by the contact of the arterial blood.

(This is a fair example of the trash that is far too readily admitted into many of the foreign medical journals, as well as into the communications addressed to the Academies on the Continent. When will these would-be physiological discoverers cease their bloody butchering experiments?—as if Nature would ever reveal her secrets to those who, with barbarous ignorance, mutilate and deform her most marvellous works!

---

### Spontaneous Rupture of the Abdomen after the Cæsarian Operation.

A woman, 37 years of age, who had been afflicted for 13 years with chronic rheumatism and softening of the bones, but who, notwithstanding, had given birth to eight children, again became pregnant, and was this time obliged to submit to the Cæsarian operation,—which was most happily attended with complete success. Between 14 and 15 months after this date, she fell once more into the family-way. In the seventh month of her gestation, she began to experience continued pain in the abdomen, which was then so pendulous that it looked like a large pouch or sac, hanging down in front, and resting on the upper part of the thighs: the gastro-hysterotomic cicatrix was visible on the surface of this depending pouch. The labour-pains became more and more violent, without effecting any good; by the 5th day, her strength appeared to be entirely exhausted.

On making an examination *per vaginam,* the outlet was found to be contracted to the smallest possible dimensions, so that the finger could not reach to the cervix uteri. The medical men in attendance were of opinion that rupture of the uterus had taken place: but, as the fœtus was dead, and as the condition of the mother was most precarious, it was deemed most advisable to leave the case to nature. About ten days subsequently, the cicatrix of the abdominal section opened, and gave issue to a fœtus, which was then in a state of putrefaction, with its appendages. Ultimately the woman recovered.—*Annales de Medicine de Gand.*

(Truly, some of these Flemish women have marvellous powers of endurance, and an extraordinary tenacity of life.)

---

### Quinine against obstinate Hiccup.

Among the many anomalous, and not unfrequently very obscure, results of miasmatic influence, are various affections of the digestive organs. Of these, Hiccup is a most troublesome, and, occasionally, a very intractable, one. In some cases, this gastric disorder has been observed to exhibit a distinctly intermittent character, and to be associated with the existence of other phenomena which are usually attributed to the operation of the same morbific cause. M. *Mendiere* has recently published, in the pages of the Revue Medicale, several cases of this sort, in which the distressing symptom of Hiccup was promptly and decisively cured by the free use of Quinine, after it had resisted every other mode of treatment. He has used the same remedy with good results in many cases of severe Cardialgia.

(The addition of a few drops of tinct. opii to the quinine will greatly enhance its efficiency in such cases. What an admirable remedy Opium is in almost all the Neuroses, provided the secretions are healthy at the time! The difficulty lies in knowing how to *time* its administration, and regulate its dose. We have usually found the Liquor Opii Sedativus the best form in which to exhibit it: this may be given in a bitter infusion, to which Ammonia may generally be added with advantage at the same time.)

---

### TREATMENT OF PLETHORA.

The treatment of Plethora is often not nearly so easy as that of Anæmia. In many cases it will not suffice merely to abstain from animal food, and to drink large quantities of simple cooling beverages, in the hope of attenuating and impoverishing the condition of the blood. Then, again, the effects of blood-letting are generally only transitory; and, moreover, the very loss of blood seems not unfrequently to induce a more active proportionate formation of it. On the whole, the use of saline laxatives, and of the Hydrochlorate of Ammonia (sal. ammoniac.) seem to be the most useful means that can be employed for the relief of Plethora, when it gives rise to inconvenient symptoms.

Dr. *Lheritier*, in his recent treatise on Pathological Chemistry, informs us that he has found that the proportion of the red globules in the blood of rabbits was decidedly modified by the internal use of this salt, in the course of two or three weeks.

(The Nitrate of Potash has similar effects; so also have the alkaline subcarbonates, and the Liquor Potassæ itself. Perhaps the latter is, on the whole, the most efficient impoverisher of the blood, provided also the diet is spare and not too nutritious, and all malt-liquors are avoided.)

---

### USEFUL HINT TO HOSPITALS.

"In the hospital at Frankfort, I noticed a peculiarity in the make of the mattrasses which deserves to be noticed. They consist of three portions or compartments, which are perfectly joined into one when the bed is made; but which are readily separated from each other, so that any part may be removed and cleaned at a time without the others. As a matter of course, it is the middle one that generally requires to be so treated. By this simple arrangement, the mattrasses may be, at a very small expense, always kept in cleanliness and order."

---

### ON THE PROTECTIVE INFLUENCE OF VACCINATION.

The general conclusions, drawn by Dr. *Retzius* of Stockholm from his observations of small-pox and the effects of vaccination in Sweden, are these: "The protection, afforded by vaccination from the close of the second year of life against the contagion of the Variolous poison, usually lasts unimpaired to the end of the thirteenth year or so: after this period it begins to lose its effect, and gradually becomes more and more uncertain on to the twentieth or twenty-first year of life. For the next four or five years, the disposition to the small-pox seems almost to have recovered its original integrity; and this state of liability continues unimpaired up to the age of forty years or so. At about this epoch of life, it begins to approach nearer and nearer to the limit of its existence—which it reaches, in the majority of cases, about the fiftieth year,—the period when the general revolution of the human body commences to take place."

(The practical inference to be drawn from these remarks is the propriety of repeating vaccination in about thirteen or fourteen years after its first performance. This advice is in accordance with the observations of the most experienced practitioners: it would be well, if it were more generally acted upon.)

---

### A Nut for the Ultra-Phlebotomists.

"I have seen," says *Bordeu*, that truly *spiritual* and lively writer, "a physician who put no bounds to his fondness for bleeding. If he had bled a patient thrice, he repeated it once more, for the good reason that there are four divisions of the world, four seasons in the year, and four cardinal points in the compass; after the fourth bleeding, a fifth was required, because there are five fingers to each hand; to the fifth he added a sixth, for that God created the world in six days; six!—oh! there must be seven, since the week has seven days, and Greece had seven Sages; an eighth is necessary to make the number even; and a ninth, *quia* * * * *numero Deus impare gaudet.*"

An amusing anecdote is told of *Barthez*, another celebrated physician of last century and cotemporary of *Bordeu*. During the excitement of the French Revolution, his house was assailed by the mob, in consequence of his having published a pamphlet in vindication of the nobility. He presented himself at his door without fear, exclaiming to the rabble, "you may break my windows, but you cannot touch my arguments." He had a bitter enemy in the person of *Linguet*, a turbulent sarcastic lawyer of the day, who, in a satirical poem, addressed him as a

Ministre de la mort, tyran de la nature,
Assassiner par art, guerir par conjecture.

---

### Consecutive Effects of Excision of the Lower Jaw.

In consequence of the division of the muscles, which attach the tongue to the jaw, there is a tendency to such a retroversion of this organ into the pharynx as to obstruct, more or less completely, the admission of the air into the *rima glottidis*. This evil is to be prevented by passing a ligature through the *frænum linguæ* and keeping the tongue forward. In some cases this retroversion is a work of very gradual occurrence; so that the breathing of the patient becomes more and more impeded, and life is extinguished by a process of slow asphyxia produced by the displacement of the tongue backwards, and the consequent obstruction of the pharynx. This seems to have been the occasion of death in a case of M. *Gerdy's*, in which the patient sunk on the ninth day after the operation, without any very evident cause. M. *Vidal* compares the death in this patient with that which is apt to occur after the operation of tracheotomy, when the artificial opening in the air-pipe is not kept sufficiently open: life becomes very gradually extinguished, and the patient dies tranquilly from a process of genuine, but very slow, asphyxia. He therefore strongly urges the necessity of paying constant attention to this circumstance.

He mentions, also, another of the subsequent and, so to speak, *chronic* effects of removing the lower jaw—viz: the lesion in the functions of digestion and the circulation, occasioned by the contraction of the laryngo-pharyngeal orifice: a state of chlorosis or anœmia is apt to be induced, and this has proved fatal.—*Annales de la Chirurgie.*

---

### Fatal Hæmorrhage from a Varix of the Vulva.

In a late number of the Berlin Medical Gazette, we find the following case related:—

A woman, pregnant of her fifth child, had, for a length of time, been annoyed with a large swelling on the left labium. One evening, immediately after going to bed, she was suddenly seized with a profuse hæmorrhage from the vulva. The midwife was accordingly summoned to her assistance; but she, finding the alarming state of the woman, sent off at once for Dr. *Hesse.* On his arrival, the patient was already in the agonies of death: the bed and floor were covered with dark coagulated blood. On examination, the orifice of the vagina was found closed, and its canal quite dry. The Cæsarian operation was immediately peformed; but the child was dead. The uterus was quite empty of blood; and the placenta adhered over its entire extent. No distinct tumor was now visible on the vulva, although the labium was still flabby and voluminous. When it was pressed, a jet of dark blood flowed out from a wound that was not more than half an inch in extent. This wound led backwards, in the direction of the perineum, to a cluster of varicose veins, one of which had burst.

### Treatment of Chronic Catarrhal Ophthalmia.

M. *Reveillé-Parise* strongly recommends the decoction, or a strong infusion, of Rhatany root, as a lotion with which the affected eyes are to be bathed. Besides acting as an astringent, this remedy seems to have some other mode of operation; for we do not find that similar preparations of oak-bark or of gall-nuts—although both of these contain a large proportion of tannin—are equally efficacious, as Collyria in the Ophthalmia alluded to. The application should be used lukewarm, and a few drops of Goulard's Extract may be added to it, if deemed proper.

### Large Doses of Nitre in Inflammatory Disease.

M. *Desportes* (Bull. de Therapeutique) has written a long paper with the view of pointing out the powerful antiphlogistic or contra-stimulant properties of Nitre, when administered in full and frequent doses, in a variety of the Phlegmasiæ. In Bronchitis and other thoracic diseases, although often inducing nausea and consequently a depression of the circulation, it is not so decidedly useful as in Acute Rheumatism and Gout: in many cases, however, of Hæmoptysis it is a truly admirable remedy.

(There is nothing new in the free exhibition of nitre or salt-petre as a remedy in the treatment of the phlegmasiæ; although some of the French writers, in the present day, seem to suppose that they are the discoverers of its virtues. The readers of *Sydenham,* and other writers of his age, know well how largely it was used by them. Like every other good remedy, it is liable to be abused; and certainly M. *Desportes* and M. *Martin Solon* are guilty of egregious blundering, when they fancy that Rheumatism, for example, may be most quickly and effectually cured by *monstre* doses of nitre. This is one of the many, not only silly but hurtful, results of the ignorant experimenting spirit of Modern Medicine.)

